# Evolution of selfish multicellularity: collective organisation of individual spatio-temporal regulatory strategies

**DOI:** 10.1186/s12862-023-02133-x

**Published:** 2023-07-19

**Authors:** Renske M. A. Vroomans, Enrico Sandro Colizzi

**Affiliations:** 1 Informatics Institute, University of Amsterdam, Amsterdam, Netherlands; 2 Origins Center, Groningen, Netherlands; 3 Sainsbury Laboratory, University of Cambridge, Cambridge, UK; 4 Mathematical Institute, Leiden University, Leiden, Netherlands

**Keywords:** Evolution of multicellularity, Evolution of regulation, Computational modelling

## Abstract

**Background:**

The unicellular ancestors of modern-day multicellular organisms were remarkably complex. They had an extensive set of regulatory and signalling genes, an intricate life cycle and could change their behaviour in response to environmental changes. At the transition to multicellularity, some of these behaviours were co-opted to organise the development of the nascent multicellular organism. Here, we focus on the transition to multicellularity before the evolution of stable cell differentiation, to reveal how the emergence of clusters affects the evolution of cell behaviour.

**Results:**

We construct a computational model of a population of cells that can evolve the regulation of their behavioural state - either division or migration - and study both a unicellular and a multicellular context. Cells compete for reproduction and for resources to survive in a seasonally changing environment. We find that the evolution of multicellularity strongly determines the co-evolution of cell behaviour, by altering the competition dynamics between cells. When adhesion cannot evolve, cells compete for survival by rapidly migrating towards resources before dividing. When adhesion evolves, emergent collective migration alleviates the pressure on individual cells to reach resources. This allows individual cells to maximise their own replication. Migrating adhesive clusters display striking patterns of spatio-temporal cell state changes that visually resemble animal development.

**Conclusions:**

Our model demonstrates how emergent selection pressures at the onset of multicellularity can drive the evolution of cellular behaviour to give rise to developmental patterns.

**Supplementary Information:**

The online version contains supplementary material available at 10.1186/s12862-023-02133-x.

## Background

The evolution of multicellularity is a major transition in individuality, which occurred multiple times across the tree of life [1–3]. These transitions were likely driven by an initial increase in cell-cell adhesion [4] - as also shown by *in vitro* evolution experiments [5, 6], leading to cluster formation by aggregation or by inhibition of cell separation [3, 7–9]. The unicellular ancestors of these nascent multicellular organisms exhibited complex behaviour and were capable of switching to different phenotypes in response to changes in the environment [10]: a form of reversible differentiation. When adhesion evolved, the newly multicellular aggregates consisted of complex cells that could exploit their pre-existing ability to differentiate in this new biotic context. Eventually the genetic toolkit organising differentiation gave rise to the developmental program of complex multicellular organisms, with stable cell differentiation organised through spatio-temporal pattern formation [3, 11, 12].

Computational models have shown that an increase in cell adhesion at the onset of multicellularity greatly impacts the evolutionary dynamics of cells because of the emergent properties of the cluster. This can be an ability to sense the environment [13, 14], or the emergence of a life cycle, alternating between aggregation and unicellularity [15] which can be driven by environmental fluctuations [16]. Once adhesion has resulted in cluster formation, the spatial structure of the newly-formed cluster affects the survival of the cells within it, and can promote cell specialisation through structured interactions [17, 18]. Conversely, cell specialisation can promote cluster formation to resolve metabolic or fitness trade-offs [19–21] and can aid the formation of stably differentiated patterning in the multicellular tissue [23, 24]. Taken together, computational models show that the transition from a unicellular to a multicellular life cycle was shaped both by spatial structuring arising from cell adhesion and by the regulation of cell behaviour.

During the development of extant multicellular groups, the precise positioning of different cell types is achieved through the interplay between spatial structure and cell behaviour. This interplay can be found in various multicellular lineages, ranging from prokaryotic biofilms to animals and plants, and may have profound consequences for the evolution and organisation of multicellularity. In simple computational models without gene expression regulation, this interplay resulted in the evolution of proto-developmental dynamics in response to graded toxic environments [25], and in genome structuring to organise cell differentiation in colonies competing for resources[26]. We construct a spatially extended computational model to investigate how the evolution of adhering cell clusters impacts the evolution of cell behaviour regulation during the first steps of the transition to multicellularity - from the ecology of unicellular populations to that of nascent multicellular organisms.

In the model, a population of spatially embedded cells has to find resources to survive. Cells have evolvable adhesion proteins, allowing for a spectrum of adhesion strength, and an evolvable regulatory network which determines their behaviour in response to a seasonally changing environment. Through their regulatory network, cells decide when to divide, and when to migrate towards the resources, allowing for various survival strategies that are characterised by one or multiple phenotypic switches between these two states.

We find that the evolved strategy depends on whether adhesion can co-evolve, especially when the environment imposes a high selection pressure. When adhesion cannot evolve, competition between cells is dominated by reaching the resources first – they have to navigate the abiotic environment to find the resources by themselves. When adhesion can evolve, cells can perform collective migration. This lowers the pressure to reach resources quickly, because cells carry each other to the peak of the gradient [14]. Cells then compete to divide earlier and more often, maximising their own reproductive success within the multicellular group. This shows that selection on cell behaviour becomes dominated by the newly established biotic environment. Thus, even within the simple context of our model, we observe a complex evolutionary transient that involves intricate feedback between group-level properties (collective migration), and individual-level competition for survival and reproduction. We call the evolved group dynamics “selfish multicellularity” to emphasize that the regulatory strategies, as well as multicellularity [14], evolve in the model because they benefit individual cells. We observe that the interactions between cells within clusters, and between cells and the environment, lead to an emergent coordination of migration and division which are reminiscent of developmental processes.

## Results

### Model setup

We construct a model of a eukaryotic organism which can evolve its life cycle to adapt to a periodically fluctuating environment. We draw inspiration from a recent reconstruction of the unicellular ancestor of animals [10], however our simple model set-up overlaps considerably with the life cycle of facultative and obligatory multicellular eukaryotes like volvocines, choanoflagellates and dictyostelid slime moulds, which exploit various forms of collective behaviour to find resources [27, 28]. We assume that adhesion between cells can evolve through a ligand and receptor system [14], and that cells migrate to resources for survival via chemotaxis.

**Fig. 1:**
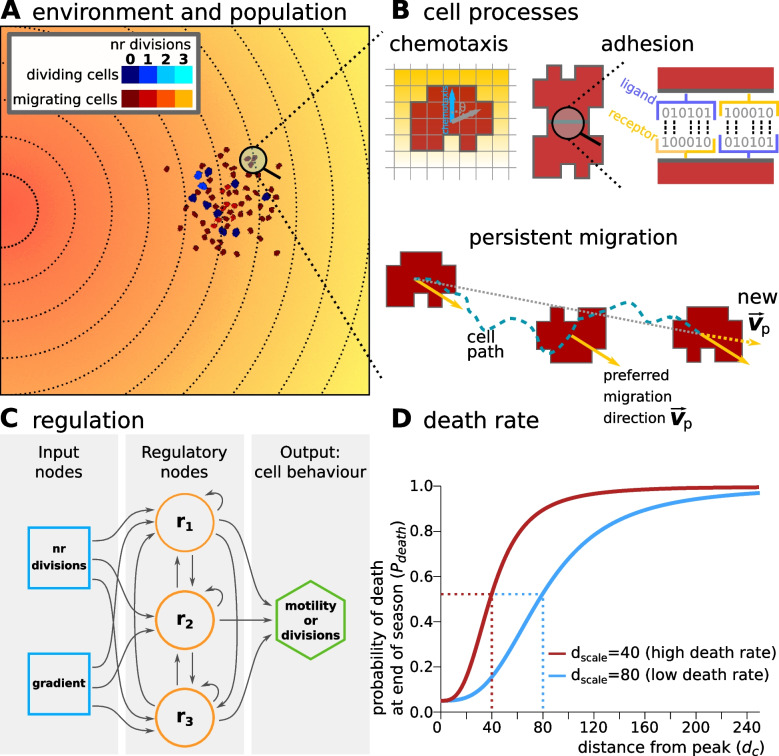
Model description. **A** The environment in which cells have to survive contains a chemoattractant gradient (lines and colour indicate equal amounts of chemoattractant). **B** Cells can sense the chemoattractant in the lattice sites that correspond to their own location, and move preferentially in the direction of perceived higher concentration (the blue arrow). Adhesion between two cells is mediated by receptors and ligands (represented by a bitstring, see Methods). The receptor of one cell is matched to the ligand of the other cell and vice versa. The more complementary the receptors and ligands are, the lower the J values and the stronger the adhesion between the cells. Persistent migration is implemented by endowing each cell with a preferred direction of motion $\textbf{v}_p$. Every $\tau _p$ MCS, this direction is updated with a cell’s actual direction of motion in that period. **C** Cells have a simple evolvable regulatory network to determine whether to migrate or divide at any given time (with max. 3 divisions per season). The network receives as input the number of divisions the cell has already done, and the concentration of the gradient. The activation threshold ($\rho _{i}$) of each node *i* and strength of interaction ($w_j$) of node *j* on node *i* can evolve to have a different effect on the state $S_i$ of the node. **D** Probability for a cell to die at the end of a season, as a function of its distance to the peak $d_c$ (in lattice sites). $d_{\text {scale}}$ determines the distance at which the probability is half-maximal

We implement a 2D hybrid Cellular Potts Model (CPM) [29–31] for the cellular dynamics, using a square lattice for the cell population and a lattice of the same dimensions for a chemoattractant signal (Fig. 1A). CPM has been extensively used to model many aspects of embryonic development [32–34], since it endows cells with an explicit size and shape, allowing for both subcellular resolution and deformation, as well as cell level properties such as adhesion and migration. We have previously shown that CPM is very suitable to modelling uni-cellular eco-evolutionary dynamics at the transition to multicellularity [14].

In the current model, cells can adhere to each other based on the ligands and receptors that they express on their membrane (Fig. 1B). The greater the complementarity between the ligands and receptors of two cells that are in contact, the stronger their adhesion. This is translated to the cell-cell and cell-medium adhesion energy in the CPM (respectively $J_{\text {c,c}}$ and $J_{\text {c,m}}$, see Methods). The adhesion strength resulting from $J_{\text {c,c}}$ and $J_{\text {c,m}}$ is quantified by the surface tension $\gamma =J \left( \sigma _1, \sigma _{\text {medium}} \right) -\frac{J \left( \sigma _1, \sigma _2 \right) }{2}$. Cells adhere to each other when $\gamma >0$ (i.e. with lower contact energy), while they preferentially interact with the medium - and thus do not adhere - when $\gamma <0$ ($\gamma ~=0$ corresponds to neutral adhesion). In some simulations, the receptors and ligands can evolve, thereby changing the strength of adhesion between cells. In simulations where adhesion cannot evolve, we set $J_{\text {c,c}}$ and $J_{\text {c,m}}$ such that the surface tension $\gamma$ between cells and the medium is negative, resulting in no adhesion.

Cells can be in one of two states: migratory or dividing, which are mutually exclusive phenotypes in our model (due to conflicting use of the cytoskeleton). When the *in silico* cells are migratory, they perform a persistent random walk and chemotaxis towards higher local concentrations of the chemoattractant signal (Fig. 1B). The chemoattractant is deposited as a gradient with a peak on one side of the lattice; this gradient is steep enough that cells are able to migrate to the peak individually. The location of the peak changes to a random side of the lattice (up, down, left or right) at the start of each new season. Dividing cells are stationary and do not react to the chemoattractant. When cells have been in the “dividing” state continuously for 10000 CPM update steps (Monte Carlo Steps, MCS – see Methods), they slowly start increasing their size over another 20000 MCS. Cells divide when they have grown to twice their original size.

We assume that cells have limited resources and that they do not acquire more over the course of a season, so that cells can divide a maximum of three times during each season. Thus, a season can be seen as the “famine” part of a “feast-and-famine” cycle. The “feast” part instead is implicit: cells that survive the end of the season simply regain enough resources to divide again three times during the next season.

The state of a cell is determined by an evolvable gene regulatory network (GRN), modeled as a Boolean network with a fixed architecture, loosely based on [35] (Fig. 1C). The network receives two inputs: the average local concentration of the chemoattractant at the cell’s location, and the number of times the cell has already divided. It has three regulatory genes that process the input and determine the state of the output gene. The state of the output gene in turn determines cell state: migratory or dividing (see Methods). When a cell has performed the maximum number of divisions in a season, it cannot divide anymore even when the state of its output gene dictates a dividing state. Instead, the cell is in a quiescent state in which it is neither dividing nor migrating. When a cell divides, the daughter cell inherits the receptors and ligands for adhesion and the GRN architecture; the state of all genes in the networks of both cells is reset to 0. In one of the daughter cells, mutations can happen in the strength of the gene regulatory interactions in the GRN and in the activation threshold of each gene.

Each season lasts a fixed number of MCS, so that cells have a fixed amount of time to divide and migrate. Different simulations can have seasons of different lengths. Cells that are closer to the peak of the chemoattractant gradient at the end of the season have a higher probability to survive into the next season than cells that are further away (Fig. 1D). At the start of each new season, the division counter in each cell, and the states of the genes in their GRN, are reset to 0.

### Short seasons and high death rates select for polarised regulatory strategies

**Fig. 2:**
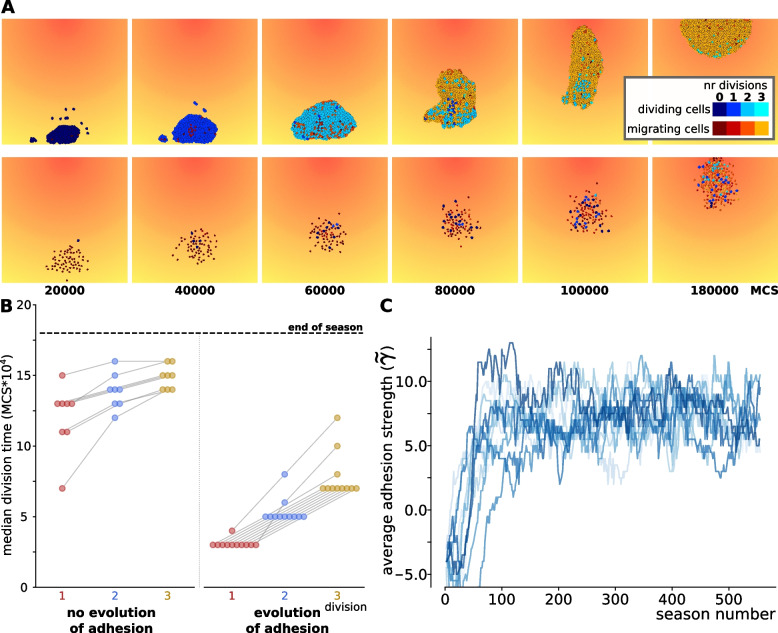
Short season duration and high death rate: either division-late or division-early evolve depending on whether adhesion co-evolves. **A** Snapshots of the population over the course of one season, in two simulations where different regulatory strategies evolved; one with and one without evolution of adhesion (season length 180000 MCS). Colours denote the state of the cell (dividing=blue, migrating=red/yellow; lighter colours indicate larger number of cell divisions). **B** Evolution of adhesion in 15 independent simulations with high death rate and short seasons. A greater median $\gamma$ (calculated from the interfacial energy between cells and with the medium, see Methods) indicates stronger adhesion between cells. **C** The median timing of cell divisions of the last 10 seasons in simulations with short seasons and high death rate, either with or without evolution of adhesion. Lines between dots connect values belonging to the same simulations (see all results in [Additional file 7])

We assessed the effect of season duration, seasonal death rate and evolution of adhesion on the evolution of cells’ regulatory strategy. We ran 15 simulations for each combination of season duration (short, intermediate or long), seasonal death rate (low or high), and the possibility of evolving adhesion, as summarised in Table 1. Each simulation was initialised with a starting population of cells possessing random GRNs, which could evolve over subsequent seasons. For milder conditions (lower death rates and longer seasons), populations in all 15 simulations could evolve viable strategies, in which cells switch at least once between migration and dividing (Fig. 2A). This led to a large population capable of reaching the peak of the chemoattractant gradient before the end of the season. In the simulations with intermediate season duration, higher death rates caused some populations to go extinct because cells were unable to evolve such switching. Under the harshest conditions (high death rates and very short seasons), extinction occurred in 4 to 7 simulation replicas (Table 1).

**Table 1:** Number of surviving populations (out of 15)

season length	death rate	No adhesion	adhesion
500000 MCS	low	15	15
	high	15	14
250000 MCS	low	15	15
	high	12	12
180000 MCS	low	13	14
	high	8	11

low: $$d_{\text {scale}}=80$$; high: $$d_{\text {scale}}=40$$

We found that a spectrum of strategies evolved in the different simulations, which could be distinguished by the timing of their divisions: early in the season, late, or somewhere in between. When cells divided very early in the season (a “division-early strategy”), they typically performed all three divisions first and then switched to the migratory state (Fig. 2A, top row; additional videos [Additional files 1, 2]). Different decision rules evolve to execute this strategy [Additional file 3A,B]. For instance, the network can evolve to count cell divisions and switch to a migratory state after the last division. In other division-early strategies, cells switch from migration to the dividing state at specific chemoattractant concentrations. In “division-late strategies”, the chemoattractant concentration was more often used as a cue, with cells starting out in a migrating state until they reached a particular concentration of the chemoattractant, performing a division, and then migrating further before dividing again at a higher chemoattractant concentration (Fig. 2A, bottom row; [Additional file 3C]; one season shown in additional videos [Additional files 4, 5]). Intermediate strategies involved cells migrating a shorter distance before performing the first division. Populations with a division-late strategy remained smaller compared to division-early strategies, because fewer cells completed all three divisions during the season [Additional file 6].

For mild conditions (longer seasons of 500000 MCS, lower death rates of $d_{\text {scale}}=80$) a broader range of strategies evolved in the different simulations, regardless of the presence or absence of adhesion evolution (all division times shown in [Additional file 7]). Simulations with shorter seasons (250000 or 180000 MCS) and higher death rate $d_{\text {scale}}=40$ led to progressively more polarised strategies. In simulations where adhesion could not evolve, we found more “division-late” solutions (Fig. 2B, left; [Additional file 7], while in simulations where the receptors and ligands for adhesion were allowed to evolve, we found more “division-early” solutions (Fig. 2B, right). In the latter simulations, the population also rapidly evolved adhesion and became multicellular (Fig. 2C), resulting in a single, large cluster of cells. Multicellularity evolves because adhering cell clusters migrate more efficiently than individual cells, and can displace non-adhering cells at the peak [14]. These results do not change when cells have a different maximum number of divisions per season [Additional file 8], representing a smaller or larger amount of resources. When the maximum number of divisions per season is very high however (seven or above), the entire grid can become filled with cells and no migration is required to reach the peak; then, cells switch to a “division-only” strategy under all conditions. The results are also independent of the precise shape of the function that determines the probability of dying at the end of the season [Additional file 9]. In short, these results suggest that the evolution of adhesion strongly affects the selection on regulatory strategy, particularly under high selection pressure.

### Multicellularity selects for the opposite regulatory strategy from unicellular organisms

Under harsh conditions, cell populations that could evolve adhesion, concomitantly evolved the opposite regulatory strategy from populations that remained unicellular. We hypothesise that, as adhesion evolves, cells in the nascent multicellular group experience a novel selection pressure stemming from the new biotic environment, that results in a division-early strategy. Similarly, a division-late strategy might evolve as a consequence of loss of adhesion, when cells revert to a unicellular state. To test this hypothesis, we assess the evolutionary stability of the two regulatory strategies - division-early and division-late - under the opposite adhesion regime.

We selected 4 individuals from different high-death rate, short-season simulations that were evolved without adhesion and let them evolve their adhesion strength. 4 individuals evolved with adhesion were used for simulations without adhesion ($\gamma =-4$). Each of these individuals was used to start a new population in 5 independent simulations.

**Fig. 3:**
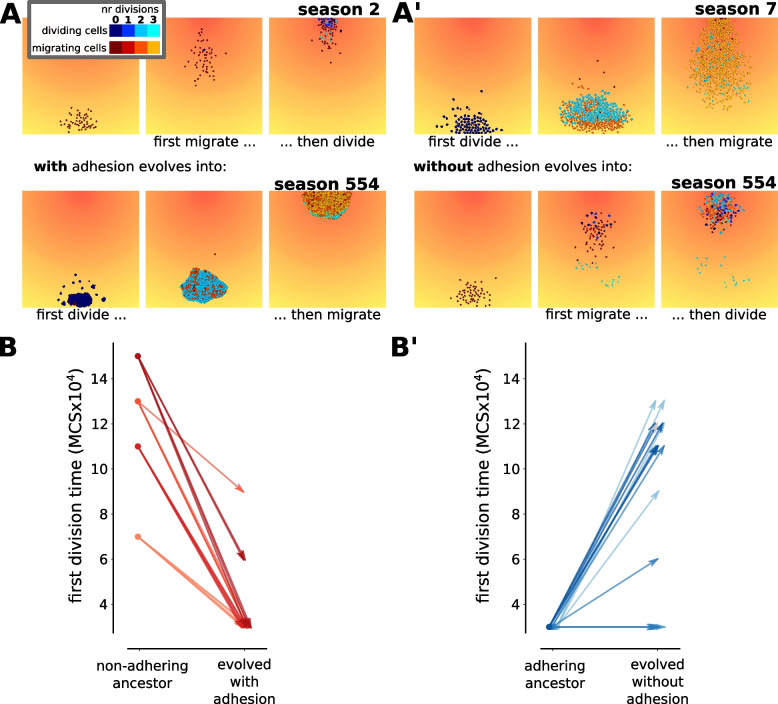
When the adhesion capacity of a population is switched, regulation evolved to the opposite strategy. **A-A’** Snapshots of a simulation from a simulation with a division-late (**A**) or a division-early (**A’**) ancestor that evolved towards the opposite strategy due to a switch in adhesion possibility (**A**: switch from simulation without adhesion to simulation with adhesion; **A’** vice versa.) (seasons were chosen in which cells start on the opposite side of the peak). textbfB-B’ Median time of the first division for all cells in the cluster. in the last ten seasons of the simulations (**B**: switch from simulation without adhesion to simulation with evolution of adhesion; **B’** vice versa.) The left circles in each graph indicate the division time of the ancestral strategy with which continued simulations were started. Four different ancestral strategies without adhesion were continued allowing for evolution of adhesion, and vice versa (5 independent simulations per ancestral strategy)

All 20 populations that could evolve adhesion, rapidly did so [Additional file 10]. Concomitantly, they evolved a division-early strategy, especially for their first division (Fig. 3A,B, [Additional file 11A,B]). Two factors contributed to earlier divisions: collective migration and the evolved regulation. Collective cell migration speeds up the group’s chemotaxis compared to a single cell [14], allowing cells to reach the chemoattractant concentration at which they divide at an earlier time – particularly when cells have a division-late strategy [Additional file 12]. Their gene regulation also evolved so that divisions occurred at a lower concentration of the chemoattractant gradient [Additional file 13A].

Instead, the individuals that were switched to simulations without adhesion gave rise to populations which evolved towards a division-late strategy Fig. 3A’, B’, [Additional file 11A,B’]). The main cause for this was a change in regulation: cells evolved to divide at a higher chemoattractant concentration [Additional file 13B]. We also observed that non adhering cells with a division-late strategy physically hindered each other while moving towards the peak of the chemoattractant gradient, leading to a slight delay in reaching the signal to divide compared to single cells [Additional file 12]. Although this effect was small, it contributed to the overall competition between cells in a division-late strategy.

In summary, the evolutionary simulations show a clear change in regulatory strategy in reaction to a change in adhesion (Fig. 3B-B’, [Additional file 11]). The experiments with a switch from non-adhesion to evolution of adhesion mimic the transition of a population to multicellularity. Populations transitioning from the unicellular to the multicellular state often shifted their division strategy faster (from division-late to division-early) than multicellular populations that had become unicellular (compare [Additional file 11B] with [Additional file 11B’]). In the latter set, some simulations took over 400 generations before shifting to a more division-late strategy. This suggests that, at least within the model context, transitioning towards a multicellular cell behaviour strategy is evolutionarily easier than its reversal.

### Adhesion drives selection for early divisions by lifting pressure to reach the peak

We next analyse the competition and cooperation dynamics during the transition to multicellularity in our model. We start from a division-late ancestor that is evolving cell adhesion (as in Fig. 3A), and consider two regulatory mutants: one that migrates for longer before dividing, and one that divides earlier. The first mutant could be considered more cooperative, because it foregoes replication to carry the group. The second then could be considered more selfish, because it gets carried by the group while prioritising its own replication. We consistently observe that the second strategy takes over, suggesting that it evolves due to competition within the cluster.

**Fig. 4:**
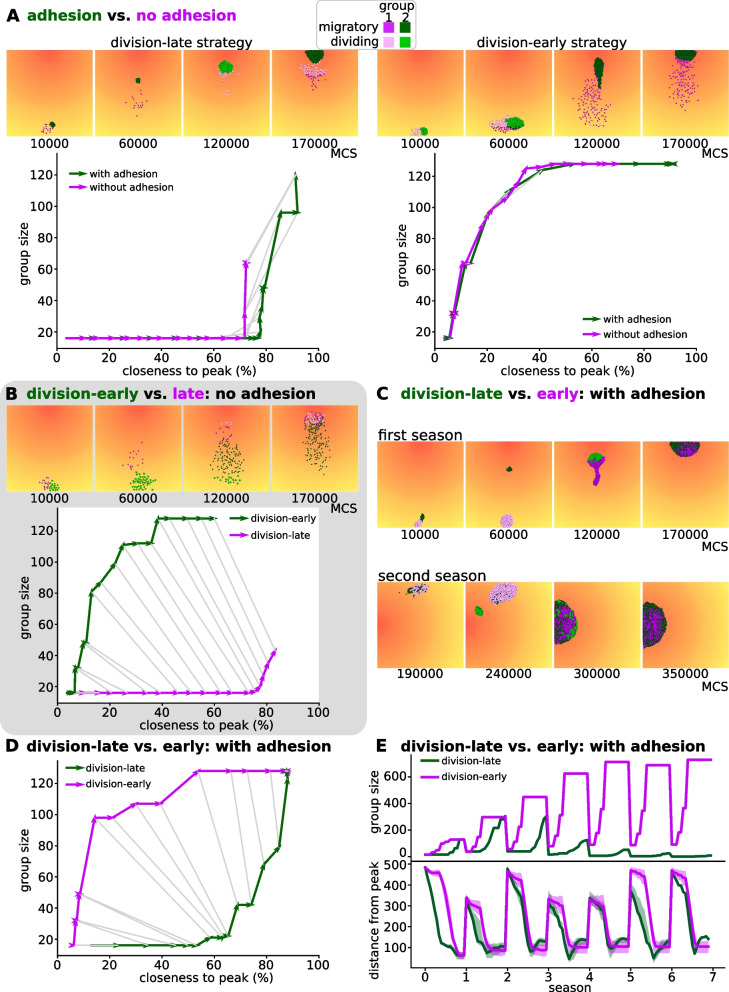
Competition experiments show that selective advantage of a regulatory strategy depends on presence of adhesion. Setup of competition experiment: two groups (with differing regulatory networks or adhesion values) are made up of 16 cells each, and are placed adjacent to each other. The simulation is then run for multiple seasons (without mutations), until one of the groups has gone extinct. In the snapshots, cells in one group are coloured green and in the other purple (lighter shade when dividing). **A-D** Snapshots show the first season of the competition simulation. Graph plots group size against median distance of cells to the peak for the first season ($0\%$=maximum distance, $100\%$=at the peak). Light-to-dark colour gradient of the line indicates time in the season, grey lines connect equal time points between the two groups. Replicates can be found in [Additional file 17]. **A** Competition between adhering ($\gamma =6$, green) and non-adhering ($\gamma =-4$, purple) cells, either both with a division-late strategy (left) or division-early strategy (right). **B** Competition between ancestral, division-early strategy (evolved with adhesion before the switch to non-adhering conditions; green in snapshots) and evolved, division-late strategy (purple in snapshots); both non-adhering. **C** Two seasons of the competition experiment between two adhering groups, one with an ancestral, division-late strategy (evolved without adhesion before the switch to a simulation in which adhesion could evolve; green in snapshots) and one with a strategy evolved after 500 seasons with adhesion (having become more division-early; purple in snapshots). **D** Group size vs. median distance from peak over the course of one season, for the competition experiment in C) **E** Time dynamics of the competition in C. Top plot shows group sizes, bottom plot shows distance to the peak. Shading indicates $25^{\text {th}}$ and $75^{\text {th}}$ percentile of population

We set up competition experiments to investigate how population dynamics cause a selective advantage for division-late strategies in unicellular populations, and for division-early strategies in multicellular groups. We placed two groups of cells with different strategies and/or different adhesion strengths next to each other at the end of the lattice opposite the peak of the gradient (Fig. 4A). Cells were allowed to migrate and divide, but no mutations could happen upon division, so that all cells in a group kept the same strategy. Cell death occurred at the end of each season as in the evolutionary simulations, and simulations were run until one of the groups went extinct.

Adhering groups always won against non-adhering groups with the same regulatory strategy (Fig. 4A). We observed that the main advantage to evolving adhesion came from adhering groups displacing non-adhering cells at the peak of the gradient. Collective chemotaxis was beneficial but had a smaller effect (Fig. 4A and see videos [Additional files 14, 15]). Therefore, adhesion evolves because it increases interference competition.

Then we investigated why the opposite regulatory strategy evolves when the adhesion regime is inverted (from division-early towards division-late when switched from adhesion to no adhesion, and vice versa). We let a division-early and a division-late population compete without adhesion (we set $\gamma =-4$). In this case, the division-late cells were closer to the peak throughout each season (Fig. 4B). The division-late strategy won because non-adhering cells exclude each other from the peak. The cells that reached the peak sooner were locked in place by the pressure exercised by the later-arriving migratory cells. (video in [Additional file 16]). However, in the first season there were significantly fewer division-late cells, indicating that this strategy sacrificed cell divisions to get to the peak sooner.

We then let a division-late group compete with division-early group, assigning both groups a high adhesion value in the competition experiment ($\gamma _{c,m}=6$). The competition dynamics of these two groups were more complex. The division-late group reached the peak of the gradient earlier than the division-early group (Fig. 4C and D). While the latter reached the peak with a larger population size, both groups had the same population size at the end of the season. Because adhesion was mediated by identical ligands and receptors, the two groups mixed freely. However, when we plotted the distance of both groups over multiple seasons, we found that the division-late group did not remain close to the peak until the end of the season, despite arriving there earlier (Fig. 4E). The videos show that the division-late cells were displaced because many still performed divisions close to the peak, and were therefore in a non-migratory state (video in [Additional file 18]). This also resulted in smaller population sizes in later seasons, as the larger mass of the division-early cells kept the division-late cells from reaching high enough chemoattractant concentrations. Thus, as adhesion increases mixing between sub-populations at the peak, a division-early strategy is more competitive because it results in larger numbers of actively motile cells.

In summary, when cells cannot evolve adhesion, their evolution is driven by competition for reaching and occupying the peak first. This leads to a division-late strategy, where cells combine the use of the abiotic environmental cues (the chemoattractant gradient) with the information on their internal state. This strategy yields a survival benefit due to lower death rates, but at the cost of fewer divisions. When cells can (and do) evolve adhesion, they optimise the use of their biotic environment, i.e., the other cells. Adhesion allows cells to reduce interference competition at the peak of the chemoattractant by increasing cell mixing, and exploit each other for migration while maximising their divisions. The evolution of multicellularity thus impacts the selection pressure experienced by the cells within the cluster.

**Fig. 5:**
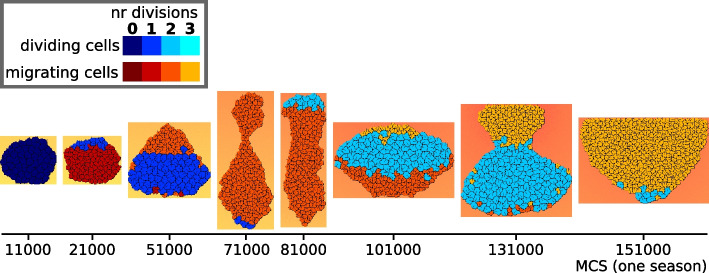
Development of genetically homogeneous cluster. Images of a developing cluster over one season. The season was started with 50 genetically identical cells with a regulatory strategy taken from from a simulation evolved with adhesion (death rate = 80, season duration = 180000). Images are centered on the cluster as it moves through the grid

Finally, the multicellular clusters often displayed patterns of cell differentiation that resembled development, with morphogenetic events such as stretching and compression of the multicellular cluster (reminiscent of convergent extension [34, 36]). This is especially clear for the dynamics of a genetically homogeneous cluster (Fig. 5, video in [Additional file 19]). Cells within the cluster reach particular chemoattractant concentrations at different times, resulting in a spatial pattern where subsets of cells are in different cell states. Unlike in complex embryonic development however, the differentiation in our simulations remains temporary in nature, with cells continually switching between a migratory and dividing state as the cluster migrates to the peak of the chemoattractant gradient.

## Discussion

In this study, we show how the evolution of adhesion changes the competition dynamics between cells that are able to sense and react to their environment. In both the unicellular and the multicellular case, cells maximise their own replication or survival, but the evolved strategy to do so depends on the abiotic and cellular context. In the model, cells evolve to regulate when to migrate towards resources and when to divide. Evolution prioritises migration in the unicellular case (when adhesion cannot evolve), because of competition to reach the resources necessary for survival. Adhesion facilitates collective migration and cell mixing, thereby lifting the selection pressure to reach the resources first. In this case, cells benefit from maximising their divisions while remaining in the migrating cluster, and from remaining as close to the peak as possible by maintaining a migratory state once there. Thus, the abiotic environment determines competition in absence of adhesion, while the biotic environment – the other cells – determines competition in the presence of adhesion. We therefore expect that at the transition to multicellularity, the regulatory program of unicellular organisms drastically changed its dynamics to suit the new (cellular) environment – potentially yielding patterning that resembles proto-development as a side effect of the process.

Current bioinformatic evidence from the holozoan relatives of animals points to a unicellular ancestor that had a complex life cycle, possibly with a multicellular stage, and a significant regulatory toolkit [10]. In fact, many of the genes necessary for coordinating multicellular development, including adhesion proteins, transcription factors and signalling genes, were already partially present in the unicellular ancestor and extant closely related unicellular species [8, 9, 37–40]. Our results show that the ancestral toolkit could have undergone rapid evolution to yield new strategies for competition within a newly multicellular context. We find that the temporal behaviour of cells, which results from their individual decoding of the environment, can already yield transient spatial patterning in the multicellular cluster. The pattern is further stabilized by the differential sorting between migrating and dividing cells. No large changes to the available genetic toolkit are required for such transient patterning, only refinements of the existing regulation.

Experiments selecting for sedimentation of yeast cells showed that two forms of multicellularity can evolve: a clonal strategy that generates small clusters through incomplete cytokinesis, and an aggregative one that generates large clusters through increased adhesion [6, 41, 42]. Aggregative groups sediment faster, but can be invaded by selfish clonal groups that exploit the increased adhesion of the aggregate [43]. Our work shows an analogous pattern, where cell clusters that prioritise migration are taken over by cell clusters that prioritise replication (Fig. 4). In the future, it would be interesting to test whether a clonal multicellular form with more complex development can evolve from an aggregative mode with such emergent properties.

In complex multicellular organisms, cell differentiation is often organised by a chemical gradient in their (tissue) environment, which is either produced by the embryo itself or via maternal factors. In contrast, external and internal cues combine to generate pattern formation in simpler multicellular organisms. For instance, in the multicellular alga *Volvox carteri*, dark-light transitions govern the differentiation of cells into somatic and germline cells, depending on their size after embryogenesis [44]. Similarly, in the multicellular (slug) phase of the slime mould *Dictyostelium discoideum*’s life cycle, cells migrate up external gradients of heat and light, causing different cell types to sort into specific regions along the body of the slug [45, 46]. Environmental cues triggering multicellularity can also be biotic: bacterial molecules induce rosette formation or swarming in the choanoflagellate *Salpingoeca rosetta* [47, 48], while some myxobacteria [49] and slime moulds migrate collectively to feed on bacteria [50].

A previous computational model showed spatial structuring of cells of different type in response to a graded environment [25], showing that this may be a general pattern. Other models have highlighted the effect of motility on the evolution of adhesion in social [51, 52] and non-social groups [14]. Our current model suggests that a transition to multicellularity may initially be driven by collective migration and increased competition within the cell cluster. We have previously shown that cell adhesion can evolve because it enables collective chemotaxis, which enhances the reproductive success of individual cells [14]. Experimental evolution with unicellular or transiently multicellular phototactic or chemotactic species may provide a test of these predictions.

Cells in the simulations form a single large cluster when cell adhesion can evolve. Within this cluster, cells keep switching between states to maximise their own growth and survival, and this competition drives the evolution of their behaviour. We call this outcome “selfish multicellularity”. We thus do not observe a complete temporal-to-spatial shift, as has been hypothesized in [53]. Multilevel competition between multiple distinct multicellular clusters may be required to select for stable differentiation, as division of labour between cells may result in more successful groups. The ensuing group-level competition may also may also reverse the evolutionary trend to selfishness, promoting more strongly cooperative strategies. In this respect, our work shows that the transition to multicellularity can be accompanied by increased competition between cells. Cooperation in the multicellular group can be a later innovation, perhaps under the control of a germ-line. Future work may show how such control may result in stable cell differentiation, as has been hypothesized before [54]. Furthermore, we show how external chemical gradients may have provided the cues for spatial pattern formation (as others have speculated [55]). The evolution of stable patterning (directed by an evolved internal gradient) may require a more evolvable genetic toolkit than currently implemented, which should expand to facilitate more complex regulation of cell-state, adhesion and cell-cell signalling. Future work may then be able to assess how the genetic toolkit used by the unicellular ancestor to regulate cell behaviour, formed the basis for stable cell differentiation and division of labour in multicellular clusters[3, 11, 12].

## Conclusions

In our previous study [14], we found that adhesion can evolve in response to an emergent selection pressure for collective migration in a noisy environment. Here, we find that such evolution of adhesion strongly determines the competition dynamics between cells, pushing it from a competition for survival to a competition for reproductive success. Adhesion therefore changes the evolution of behaviour regulation. In combination with an external chemoattractant gradient, this leads to the emergence of differentiation patterns in newly multicellular organisms.

## Methods

We model an evolving population of cells that can adhere to each other, divide, migrate and perform chemotaxis on a two-dimensional lattice containing a gradient of chemotactic signal. Cells consist of multiple lattice sites, giving them an explicit shape and volume. They also contain an evolvable gene regulatory network that senses the environment and regulates the decision to either divide or migrate (explained below). Cell dynamics on the lattice (movement, adhesion) are governed by the Cellular Potts Model (CPM) formalism [29, 30] and simulated with a Monte Carlo method. The population undergoes a seasonal culling, in which cells further from the chemoattractant source have a higher chance of dying. Then, the chemoattractant source is placed at a different position, after which the new season starts. The custom software, written in C++, can be found at [56]. Parameter values are listed in Table 2.

**Table 2:** Parameters

Parameter	explanation	Values
$L^2$	lattice size	$500 \times 500$ lattice sites
*T*	Boltzmann temperature	16 AUE
$\lambda$	cell stiffness	4.0 AUE/[lattice site]$^2$
$A_T$	cell target area	50 lattice sites
*Cell adhesion*		
$J_\alpha$	minimum J value between cells	4 AUE/[lattice site length]
$J_\alpha '$	minimum J value between cell and medium	8 AUE/[lattice site length]
$\nu$	length of receptor and ligand bitstring	24 bits
$\nu '$	length ligand bitstring for medium adhesion	6 bits
*Cell migration and chemotaxis*		
$\mu _p$	strength of persistent migration	3.0 AUE
$\tau _p$	duration of persistence vector	50 MCS
$\mu _{\chi }$	strength of chemotaxis	1.0 AUE
$k_{\chi }$	scaling factor chemoattractant gradient	5.0 molecules/[lattice site length]
*Cell division*		
$\eta _{\text {init}}$	time before cell in GRN state 1 can start growing	10000 MCS
$\eta _{\text {grow}}$	time for cell in state 1 to grow to 2x $A_T$ and divide	20000 MCS
*Evolution*		
$\tau _s$	duration of season	$18 \times 10^4$ - $50 \times 10^4$ MCS
$p_{\text {min}}$	minimum probability of dying	0.05
$p_{\text {max}}$	maximum probability of dying	1.0
$d_{\text {scale}}$	distance from gradient peak where death probability is $\frac{1}{2}$ maximum	40 or 80 [lattice site length]
$\mu _{R,I}$	receptor and ligand mutation probability	0.01 per bit, per replication
$\mu _{\omega }$	mutation probability of network parameters (*w*, *f*, $r_a$)	0.02 per parameter, per replication
$\sigma$	standard deviation of mutation size	0.05

*AUE* Arbitrary Units of Energy (see the formulation of the Hamiltonian in the Cellular Potts Model section), *lattice site* unit of area, *lattice site length* unit of distance, *MCS* Monte Carlo Step (unit of time)

### Regulation of cell behaviour

Cells have a simple evolvable regulatory network that determines their behaviour: either migrating and following chemotactic signals, or growing and dividing. The overall network architecture has been previously used to model gene regulation in micro-organisms [35], and consists of 2 sensory nodes, 3 regulatory nodes and an output node, totalling $N=6$ nodes (Fig. 1B). The sensory nodes receive the local concentration of chemoattractant (a real number between 0 and 28) and the number of times the cell has already divided. The regulatory nodes receive input from the sensory nodes and regulatory nodes, including themselves. The output node takes as input the state of the regulatory nodes, to determine whether the cell is in a migratory or dividing state. The regulatory nodes and output node are Boolean. The new state of each node *i* is calculated synchronously from the previous state of the network, as follows:

$$\begin{aligned} r_i(t+1)=\sum _jw_{j \rightarrow i} \, S_j(t) \end{aligned}$$

$$\begin{aligned} S_i(t+1) = \left\{ \begin{array}{ll} 1 &{} \text {if}\ r_i>\rho _{i}\\ 0 &{} \text {otherwise} \\ \end{array}\right. \end{aligned}$$

where $r_i(t+1)$ is the regulatory input into node *i* from nodes *j* in state $S_j$ at time *t*, which regulate *i* with weight $w_{j \rightarrow i}$. $S_i(t+1)$ is then the new state of node *i*, at time $t+1$. The new state is 1 if the value of the regulatory input $r_i$ is greater than its activation threshold $\rho _{i}$. When the node *j* is an input node, $S_j$ is the value of the input node times an evolvable scaling factor $\phi _j$; otherwise it is the Boolean state of a regulatory node (including *i* itself). Since the update is synchronous, the state of all nodes is only updated after all new node states are calculated from the old node states. For each cell, the network state is updated every 20 MCS, to even out microscopic cell fluctuations. The networks of different cells are updated at different MCS to prevent artificial synchrony between cells.

When the state of the output node is 0, the cell is in migratory mode. When the state is 1 for $\eta _{\text {init}}$ consecutive timesteps, the cell enters the dividing cell state, during which it does not migrate or perform chemotaxis. It will need $\eta _{\text {grow}}$ timesteps to grow to twice the size (by increasing target size, $A_T$, every $\eta _{\text {grow}}/A_T$ MCS) and prepare for division before it can actually divide. If, in that time, the output node becomes 0, the cell will shrink again and revert back to the migratory state, and will start the division process anew if the output node becomes 1 again. Once the cell divides, the state of all nodes is reset to 0. A cell can divide a maximum of three times per season; when the output node remains one after that, the cell is non-migratory, but won’t divide again.

Upon cell division, the regulatory network is passed to the daughter cell with the possibility of mutations. Each parameter of the network ($w_i$, $\phi _i$, $\rho _{i}$) mutates independently during cell division. Mutations occur with probability $\mu _\omega$ and change the values of these parameters by a small random number sampled from a normal distribution with mean 0 and $\sigma =0.05$.

### Cellular Potts Model

The Cellular Potts Model dynamics are implemented with the Tissue Simulation Toolkit [31], on a regular square lattice $\Lambda _1 \subset \mathbb {Z} ^2$ of size $L \times L$. A cell *c* consists of the set of lattice sites $\textbf{x} \in \Lambda _1$ with the same spin *s*, i.e. $c(s) = \{ \textbf{x} \in \Lambda _1 \mid \sigma (\textbf{x})=s \}$. The chemotactic signal is located on a second plane $\Lambda _2$, of the same size and spacing as $\Lambda _1$.

The cells move in the $\Lambda _1$ lattice due to the displacement of their boundary, arising from stochastic fluctuations. These fluctuations tend to minimise a cell’s energy, whose terms correspond to biophysically motivated cell properties ([57]). The $\Lambda _1$ lattice is updated through the Metropolis algorithm. Each Monte Carlo step (MCS), $L \times L$ lattice sites are drawn randomly. For each site belonging to the boundary of a cell, a random site from its Moore neighbourhood, $\textbf{x}$, is selected which may copy its spin value $\sigma (\textbf{x})$ into this lattice site. To speed up simulations, we used an algorithm that only considers updates for neighbouring lattice sites that have a different *s*, as implemented in [58]. Whether an attempted spin copy is accepted depends on the contribution of several terms to the energy *H* of the system, described by the Hamiltonian, as well as other biases *Y*. A copy is always accepted if energy is dissipated, i.e. if $\Delta H + Y < 0$ (with $\Delta H = H_{\text {after copy}} - H_{\text {before copy}}$), and may be accepted if $\Delta H + Y \ge 0$ because of “thermal” fluctuations that follow a Boltzmann distribution:

$$\begin{aligned} P(\Delta H, Y) = e^{\frac{-(\Delta H + Y)}{T}} \end{aligned}$$

with $T=16$ the Boltzmann temperature (in Arbitrary Units of Energy AUE), which controls the probability of energetically unfavourable copy events. The Hamiltonian consists of two terms, corresponding to cell size maintenance and adhesion:

$$\begin{aligned} H = H_\text {cell size} + H_\text {adhesion} \end{aligned}$$

The copy biases, or “work terms”, *Y* consist of terms corresponding to cell migration and chemotaxis:

$$\begin{aligned} Y = Y_\text {migration} + Y_\text {chemotaxis} \end{aligned}$$

### Cell size maintenance

Cell size $A(c) = |c(s)|$, the number of lattice sites that compose a cell, is assumed to remain close to a target size $A_T$. Deviations from the target size are inhibited by adding the following term to the Hamiltonian:

$$\begin{aligned} H_\text {cell size} = \sum _{c \text { } \in \text { } C} \lambda \left( A \left( c\right) - A_T \right) ^2 \end{aligned}$$

with *C* the set of cells and $\lambda$ representing the resistance of cells against volume changes. A cell’s target volume may grow when it prepares to divide, and is halved again upon division (see also below).

### Cell adhesion

The total adhesion energy resulting from interfaces between cells and with the medium is implemented as:

$$\begin{aligned} H_\text {adhesion} = \sum _{ \left( \textbf{x},\textbf{x}^{\text { }\prime }\right) } J \left( \sigma (\textbf{x}), \sigma (\textbf{x}^{\text { }\prime }) \right) (1 - \delta \left( \sigma (\textbf{x}), \sigma (\textbf{x}^{\text { }\prime }) \right) ) \end{aligned}$$

summing over all pairs of neighbouring pixels with different *s*: $\left( \textbf{x},\textbf{x}^{\text { }\prime } \right)$.

$\delta \left( \sigma (\textbf{x}), \sigma (\textbf{x}^{\text { }\prime }) \right)$ is the Kronecker delta which restricts the energy calculations to the interfaces.

As previously described [14], cells express ligand and receptor proteins on their surface that determine the surface energy of cell interfaces, $J\left( \sigma (\textbf{x}), \sigma (\textbf{x}^{\text { }\prime }) \right)$. Ligands and receptors are modelled as binary strings of fixed length $\nu$ (Fig. 1). Cell adhesion increases (i.e. lower *J* values) with greater complementarity between their receptors *R* and ligands *I* (i.e. larger Hamming distance $D(R,I) =\sum _{i=1}^\nu 1-\delta (R_i,I_i)$ ), where $R_i$ and $I_i$ are corresponding bits in the receptor of one cell and the ligand of the other cell, and $\delta$ is the Kronecker delta function which is 1 when the two bits match. Thus, given two cells with spin values $\sigma _1$ and $\sigma _2$ and their corresponding pairs of receptors and ligands $(R(\sigma _1),I(\sigma _1))$ and $(R(\sigma _2),I(\sigma _2))$:

$$\begin{aligned} J \left( \sigma _1, \sigma _2 \right) = J_\alpha + 2 \nu - D(R(\sigma _1),I(\sigma _2)) - D(R(\sigma _2),I(\sigma _1)) \end{aligned}$$

with $J_\alpha = 4$ so that $J \left( \sigma _1, \sigma _2 \right)$ ranges between [4, 52].

Adhesion of a cell with medium is assumed to depend only on the cell (the medium is inert, i.e. $J \left( \sigma _{\text {medium}}, \sigma _{\text {medium}}\right) = 0$ ), and in particular it depends only on a subset of the ligand proteins of a cell. This subset consists of the substring of *I*, $I_{[0,\nu \prime ]}$, which begins at the initial position of *I* and has length $\nu ^\prime$. The value of $J \left( \sigma _1, \sigma _{\text {medium}} \right)$ is calculated as:

$$\begin{aligned} J \left( \sigma _1, \sigma _{\text {medium}}\right) = J_\alpha ^\prime + \sum _{i=1}^{\nu ^\prime } F(i)I_i \end{aligned}$$

$$\begin{aligned} F(i) = \left\{ \begin{array}{ll} 4 &{} \text {if}\ i=1 \\ 3 &{} \text {if}\ i=2 \\ 2 &{} \text {if}\ i=3 \\ 1 &{} \text {if}\ 4\le i \le 6 \\ \end{array}\right. \end{aligned}$$

with $J_\alpha ^\prime = 8$ and *F*(*i*) a piece-wise defined function (a lookup table). Thus, each bit $I_i$ in $I_{[0,\nu \prime ]}$ contributes to $J \left( \sigma _1, \sigma _{\text {medium}}\right)$ with a different weight, resulting in *J* values falling in the interval [8, 20].

The strength with which cells adhere to each other depends on both the J value between the cell and the medium, and the J value of the interaction between cells. This can be represented by the $\gamma$ value, the surface tension between cells and medium:

$$\begin{aligned} \gamma =J \left( \sigma _1, \sigma _{\text {medium}} \right) -\frac{J \left( \sigma _1, \sigma _2 \right) }{2} \end{aligned}$$

When $\gamma$ is negative, cells preferentially interact with the medium and do not adhere. In simulations where adhesion cannot evolve, we set $J \left( \sigma _1, \sigma _2 \right) =36$ and $J \left( \sigma _1, \sigma _{\text {medium}} \right) =14 \rightarrow \gamma =-4$, yielding nonadhering cells. To simplify the notation in the main text, we use the subscripts c and m to refer to lattice sites belonging to cells and medium, so that $J_{c,m} = J(\sigma _1, \sigma _{medium})$ and $J_{c,c} = J(\sigma _1, \sigma _2)$, where the subscript c,c always assumes two different cells.

In a subset of simulations, the bitstrings of the receptor and ligand responsible for adhesion are mutated upon cell division. These mutations occur in only one of the two daughter cells (chosen at random) with a per-position probability $\mu _{\text {R,I}}$. Mutations flip individual bits (from 0 to 1, and vice versa).

### Cell migration

We model migration (following [59]) by biasing cell movement to their previous direction of motion $\textbf{p} \left( c\right)$: extensions of a cell are energetically more favourable when they are closer to the direction of that cell’s $\textbf{p}$:

$$\begin{aligned} Y_\text {migration} = - \mu _{\text {p}} \cos (\theta _p) \end{aligned}$$

Where $\mu _{\text {p}}$ is the maximum energy contribution due to migration, and $\theta _p$ is the angle between $\textbf{p}$ and the vector that extends from the center of mass of the cell to the lattice site into which copying is attempted. Every $\tau _p$ MCS the vector $\textbf{p}$ is updated to reflect the actual direction of displacement of the cell over the past $\tau _p$ MCS (scaled to unit) (Fig. 1). We set $\tau _p = 50$ MCS. Whether a cell migrates depends on its internal state. Dividing cells do not migrate, so their $\mu _{\text {p}}=0$. Non-dividing cells have $\mu _{\text {p}}=3.$ Note that all cells have the same $\tau _p$, but they update their vectors at different MCS to prevent them from moving synchronously.

### Chemotaxis

Individual cells are able to migrate towards the perceived direction of a chemoattractant gradient. In contrast to [14], the slope of the gradient is steeper and we removed a source of noise in the gradient signal, allowing individual cells to identify the location of the peak with ease.

The chemotactic signal is represented by a collection of integer values on a second two dimensional lattice ($\Lambda _2 \subset \mathbb {Z} ^2$, with the same dimensions as the CPM lattice), which remain constant for the duration of one season ($\tau _s$ MCS). The amount of chemotactic signal $\chi$ is largest at the peak, which is located at the center of one of the lattice boundaries, and from there decays linearly in all directions, forming a gradient: $\chi (d) = 1 + (k_\chi \frac{d_{\text {max}}}{100})*(1 - \frac{d}{d_{\text {max}}})$, where $k_\chi$ is a scaling constant, *d* is the Euclidean distance of a lattice site from the peak of the chemoattractant gradient, and $d_{\text {max}}$ is the maximum distance between the source of the chemoattractant and any lattice site in $\Lambda _2$. Non integer values of $\chi$ are changed to $\lceil \chi \rceil$ (the smallest integer larger than $\chi$) with probability equal to $\lceil \chi \rceil - \chi$, otherwise they are truncated to $\lfloor \chi \rfloor$ (the largest integer smaller than $\chi$).

A cell *s* only perceives the chemotactic signal on the portion of $\Lambda _2$ corresponding to sites on $\Lambda _1$ with that spin. We define the vector $\mathbf {\chi }(c)$ as the vector that spans from the cell’s center of mass to the center of mass of the perceived gradient. Copies of lattice sites are favoured when they align with the direction of the vector $\mathbf {\chi }(c)$, i.e. when there is a small angle $\theta _c$ between $\mathbf {\chi }(c)$ and the vector that spans from the center of mass of the cell to the lattice site into which copying is attempted (Fig. 1):

$$\begin{aligned} Y_\text {chemotaxis} = - \mu _{\chi } \cos ( \theta _c) \end{aligned}$$

where $\mu _{\chi }$ is the maximal propensity to move along the perceived gradient, and is set to $\mu _{\chi } = 1$ except for dividing cells, whose $\mu _{\chi }=0$. A uniform random $\theta _c \in [0,2\pi [$ is chosen whenever $|\mathbf {\chi }(c)|=0$, i.e. when, locally, there is no gradient (due to the chemoattractant amount at each lattice site being rounded to integer values).

### Seasonal dynamics

A population of *N* cells undergoes the cell dynamics described above for the duration of a season, i.e. $\tau _s$ MCS. Cells can divide throughout the season, and upon division will pass on their regulatory network to the daughter cell with the possibility of mutations (described above). At the end of the season, we assess the distance of each cell from the peak of the chemoattractant gradient. The further the cell, the higher its probability of being killed before the new season starts. This is implemented as:

$$\begin{aligned} P_{\text {death}}(d_c)=p_{\text {min}}+\frac{(p_{\text {max}}-p_{\text {min}})*d_c^3}{d_{\text {scale}}^3+d_c^3} \end{aligned}$$

where $p_{\text {min}}$ is the minimum probability of dying, $p_{\text {max}}$ the maximum probability, $d_c$ is the distance of cell *c* to the peak of the gradient, and $d_{\text {scale}}$ is the distance at which the death probability is at half of the maximum. The smaller $d_{\text {scale}}$, the more cells will die.

## Supplementary Information

**Additional file 1.** Division-early strategy evolved without adhesion. One season of a simulation which evolved a division-early strategy (Season duration=250000; high death rate; adhesion cannot evolve).

**Additional file 2.** Division-early strategy evolved with adhesion. One season of a simulation which evolved a division-early strategy (Season duration=250000; high death rate; adhesion can evolve).

**Additional file 3.** Assessment of evolved GRN responses in simulations with and without adhesion. **A)** We assess the steady state of the output node for all combinations of values for the input nodes (gradient concentration and number of divisions) that are possible in the simulation (with the other nodes starting at 0), and then assign that combination a color in the 2D profile, with red=migratory (output node 0), blue=dividing (output node 1). The profile can be read by starting on the bottom-left – as for a cell that has not divided yet and is far from the peak of the gradient – and following the yellow line. When the current pixel is red, the line moves up to indicate the cell migrating to a higher concentration of the gradient, and the line moves right when the pixel is blue to indicate a division (increasing the number of divisions the cell has done). **B)** Examples of GRN responses of division-early cells (evolved with the possibility of evolving adhesion). The left-most cell only counts divisions: it switches to migrating at the third division, while the others migrate until they reach a particular concentration and then switch to division. For the left-most cell, the evolved gene regulatory network is depicted, with the input nodes in light blue, the regulatory nodes in orange and the output node in green. Blue edges are activating and red edges are repressing, with the thickness of the edge indicating the weight $w$ of the interaction (weights are also indicated next to the edge). The number in the input node indicates the evolved scaling factor $\phi$; the numbers in the other nodes indicate the evolved activation threshold $\rho$ of that node. **C)** Examples of GRN responses of division-late cells (evolved without adhesion). For the first individual, the evolved gene regulatory network is depicted. The simulations shown in B and C are used to seed the simulations in which adhesion evolution is switched (Fig. 3 of the main text).

**Additional file 4.** Division-late strategy evolved without adhesion. One season of a simulation which evolved a division-late strategy (Season duration=250000; high death rate; adhesion cannot evolve).

**Additional file 5.** Division-late strategy evolved with adhesion. One season of a simulation which evolved a division-late strategy (Season duration=250000; high death rate; adhesion can evolve).

**Additional file 6.** Evolved population size. The population sizes at the end of the last ten seasons in simulations with high death rate and very short seasons (180000 MCS). Simulations are sorted by their median first division times (indicated on the x axis), and colours group data from the same simulation. With these season parameters, division-late strategies typically evolve in simulations without evolution of adhesion, and division-early strategies evolve in simulations with evolution of adhesion. The simulations shown here are used to seed the simulations in which adhesion evolution is switched (Fig. 3 of the main text). Resolution on division timing was limited to 10000 MCS.

**Additional file 7.** Evolved division timing strategies in all simulation sets. The median timing of cell divisions of the last 10 seasons in all sets of simulations, with varying season duration, death rates and with or without evolution of adhesion. Lines between dots connect values belonging to the same simulations.

**Additional file 8.** Evolved division timing strategies in simulations with a different maximum number of divisions. The median timing of cell divisions of the last 10 seasons in simulations with high selection pressure (short seasons, 180 000 MCS; and high death probability, $d_{scale} = 40$). Lines between dots connect values belonging to the same simulations. On the right, snapshots of one season in representative simulations are displayed.

**Additional file 9.** Evolved division timing strategies in simulations with different death function. A) The function that determines the probability of death at the end of the season based on the distance of a cell from the peak of the gradient. In blue the function used for the simulations in the main text; in red, a linear function that a linear function that increases until it reaches probability of death = 1, and remains constant for further distances. B) Simulations run with or without adhesion, using the linear death rate function. C) Snapshots from one season of representative simulations with a linear death rate.

**Additional file 10.** Evolution of adhesion in simulations starting from individual evolved without adhesion. Evolution of adhesion in 20 simulations, started with 4 individuals that had evolved without adhesion (5 independent simulations per individual).

**Additional file 11.** Evolution of the opposite cell regulation strategy when adhesion is switched. A) For all simulations, the evolved timing of the first division is plotted against the evolved adhesion strength. Blue: simulations that were started with an adhering ancestor, but continued evolution without adhesion ($\gamma$ fixed to -4); red: simulations started with a non-adhering ancestor that were allowed to evolve adhesion. B) Evolution of division timing in simulations started with a non-adhering ancestor. B’) Evolution of division timing over multiple seasons in simulations started with an adhering ancestor that were continued without adhesion.

**Additional file 12.** Effect of adhesion or non-adhesion on division timing. The distribution of division timings over one season for simulations with a single cell compared to simulations with 20 adhering or non-adhering cells with identical regulatory networks. For simplicity, divisions were simulated by having the cell cease migration or the same amount of time as an actual division would last, but without creating an additional daughter cell. This kept the group size the same throughout the run. There were also no mutations of adhesion or regulation. We show here two examples, one with cells evolved without adhesion, possessing a division-late strategy; and one with cells evolved with adhesion, possessing a division-early strategy. Season duration=180000 MCS.

**Additional file 13.** Evolution of gradient sensing in populations switched to opposite adhesion regime. Distributions of the chemoattractant concentration at which divisions happen, in the ancestor and the five replicate simulations evolved from that ancestor. A) Simulations seeded with individuals evolved without adhesion, continued with evolution of adhesion. B) Simulations seeded with individuals evolved with adhesion, continued without adhesion.

**Additional file 14.** Competition experiment 1. A competition experiment between a cluster of adhering cells (green) and a cluster of non-adhering cells (purple), with division-late strategy.

**Additional file 15.** Competition experiment 2. A competition experiment between a cluster of adhering cells (green) and a cluster of non-adhering cells (purple), with division-early strategy.

**Additional file 16.** Competition experiment 3. A competition experiment between a cluster of division-early ancestors (green) and a cluster of division-late descendants (purple) – both nonadhering.

**Additional file 17.** Replicates of competition experiment. Group size plotted against the median distance of cells to the peak of gradient, shown for the first season of the competition experiment (0%=maximum distance, 100%=at the peak). The graded lines with dots are the same as in Fig. 4. **a-b**) Competition between two groups with the same regulatory strategy, one adhering ($\gamma = 6$; a), the other non-adhering ($\gamma = -4$; b). **c**) Competition between ancestral, division-early strategy and evolved, division-late strategy; both non-adhering. **d**) Competition between two adhering groups, one with an ancestral, division-late strategy and one with a strategy evolved with adhesion (having become more division-early).

**Additional file 18.** Competition experiment 4. A competition experiment between a cluster of division-late ancestors (green) and a cluster of division-early descendants (purple) – both adhering.

**Additional file 19.** Developmental dynamics of homogeneous adhering cluster. One season of a simulation with a clonal cluster of initially 50 cells (i.e. all have the same regulatory strategy). The regulatory strategy was sampled from the last season of a simulation in which the population was switched from non-adhering to evolution of adhesion.

## Data Availability

The source code used for the simulations in this study is freely available from Github: [56], https://github.com/RenskeVroomans/regulation_evolution. The simulation data that were generated for the current study are available from the corresponding author on reasonable request.

## Abbreviations

CPM: Cellular Potts Model
MCS: Monte Carlo Step
GRN: Gene regulatory network
