# Supplementary figures and images for: Evolution of selfish multicellularity: collective organisation of individual spatio-temporal regulatory strategies
Source: BMC Ecol Evol. 2023 Jul 19;23:35. doi: 10.1186/s12862-023-02133-x (PMC10357660; doi:10.1186/s12862-023-02133-x)

**A**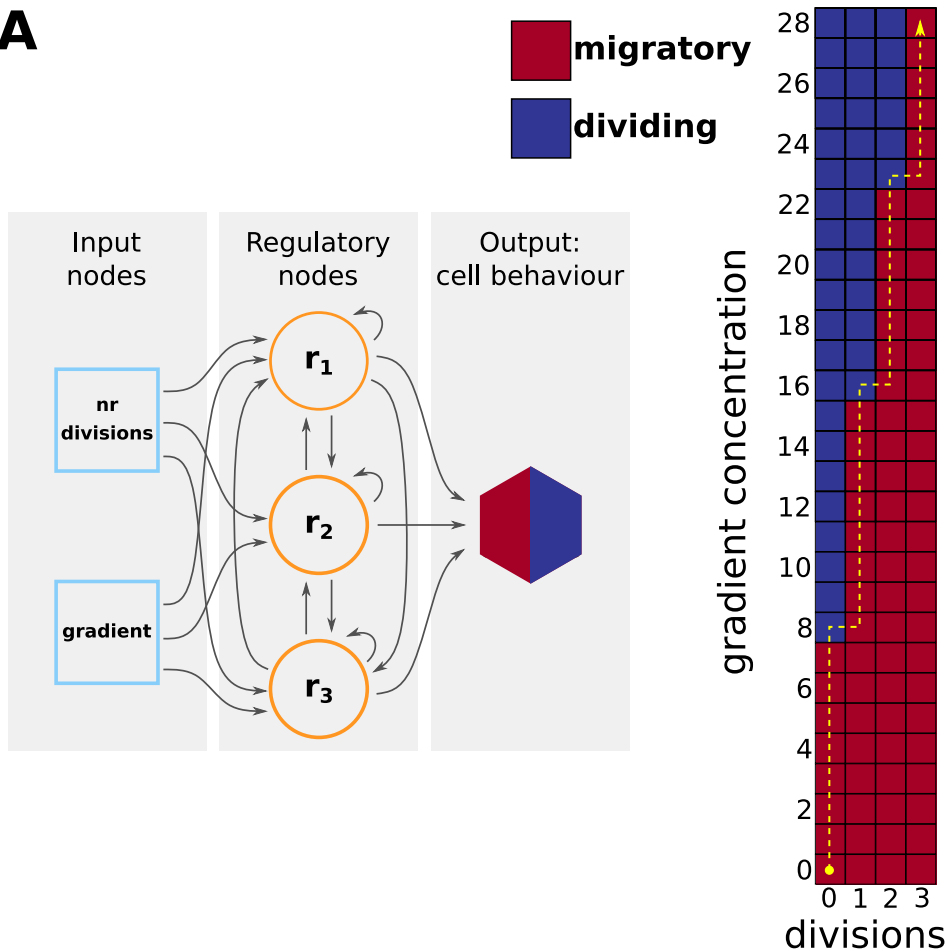**B** division-early, evolved with adhesion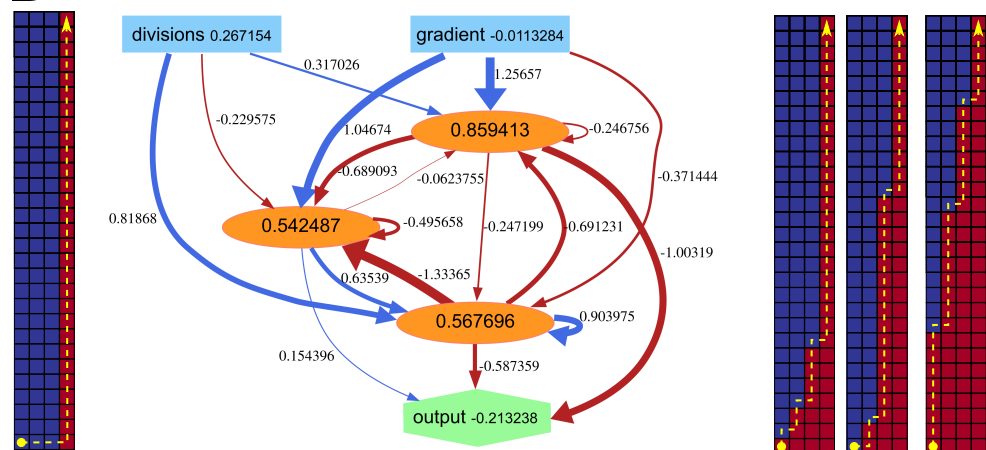**C** division-late, evolved without adhesion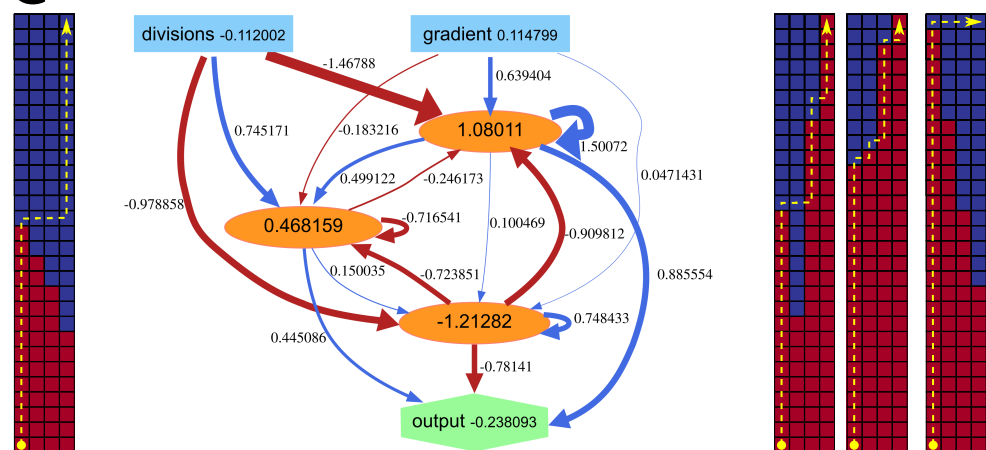

Supplement: Supplementary file 3 — Additional file 3. Assessment of evolved GRN responses in simulations with and without adhesion. A) We assess the steady state of the output node for all combinations of values for the input nodes (gradient concentration and number of divisions) that are possible in the simulation (with the other nodes starting at 0), and then assign that combination a color in the 2D profile, with red=migratory (output node 0), blue=dividing (output node 1). The profile can be read by starting on the bottom-left – as for a cell that has not divided yet and is far from the peak of the gradient – and following the yellow line. When the current pixel is red, the line moves up to indicate the cell migrating to a higher concentration of the gradient, and the line moves right when the pixel is blue to indicate a division (increasing the number of divisions the cell has done). B) Examples of GRN responses of division-early cells (evolved with the possibility of evolving adhesion). The left-most cell only counts divisions: it switches to migrating at the third division, while the others migrate until they reach a particular concentration and then switch to division. For the left-most cell, the evolved gene regulatory network is depicted, with the input nodes in light blue, the regulatory nodes in orange and the output node in green. Blue edges are activating and red edges are repressing, with the thickness of the edge indicating the weight $w$w of the interaction (weights are also indicated next to the edge). The number in the input node indicates the evolved scaling factor [file 12862_2023_2133_MOESM3_ESM.pdf]

**without adhesion**

**with adhesion**

population size

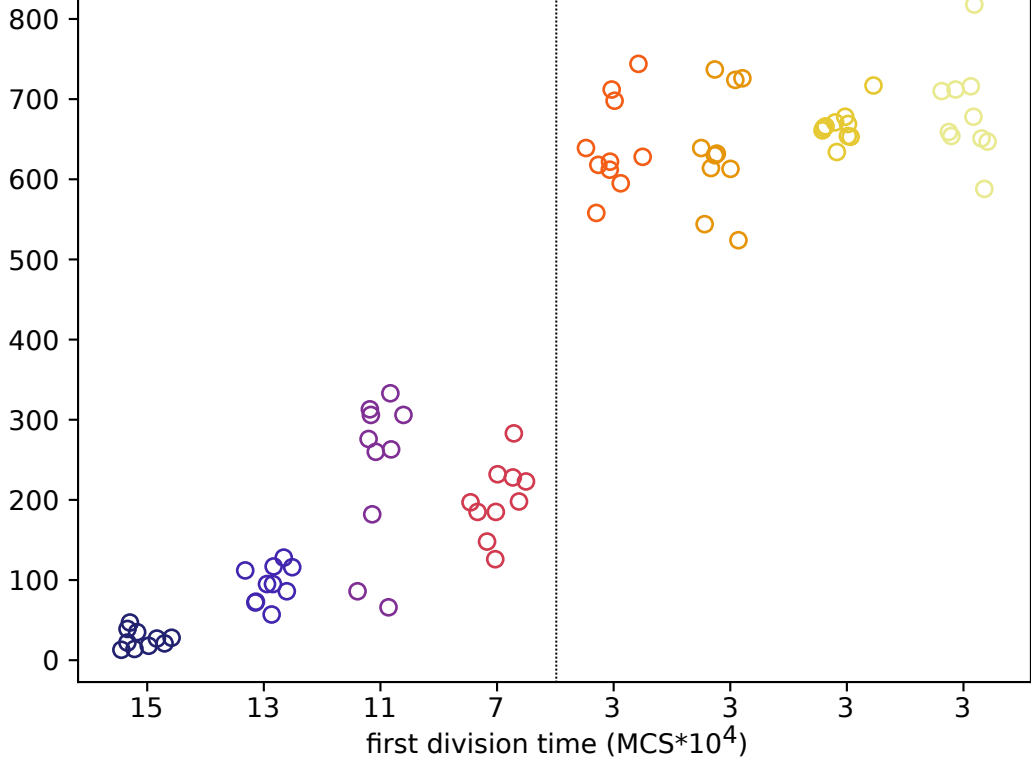

Supplement: Supplementary file 6 — Additional file 6. Evolved population size. The population sizes at the end of the last ten seasons in simulations with high death rate and very short seasons (180000 MCS). Simulations are sorted by their median first division times (indicated on the x axis), and colours group data from the same simulation. With these season parameters, division-late strategies typically evolve in simulations without evolution of adhesion, and division-early strategies evolve in simulations with evolution of adhesion. The simulations shown here are used to seed the simulations in which adhesion evolution is switched (Fig. 3 of the main text). Resolution on division timing was limited to 10000 MCS. [file 12862_2023_2133_MOESM6_ESM.pdf]

# low death rate

# high death rate

season=500000 MCS

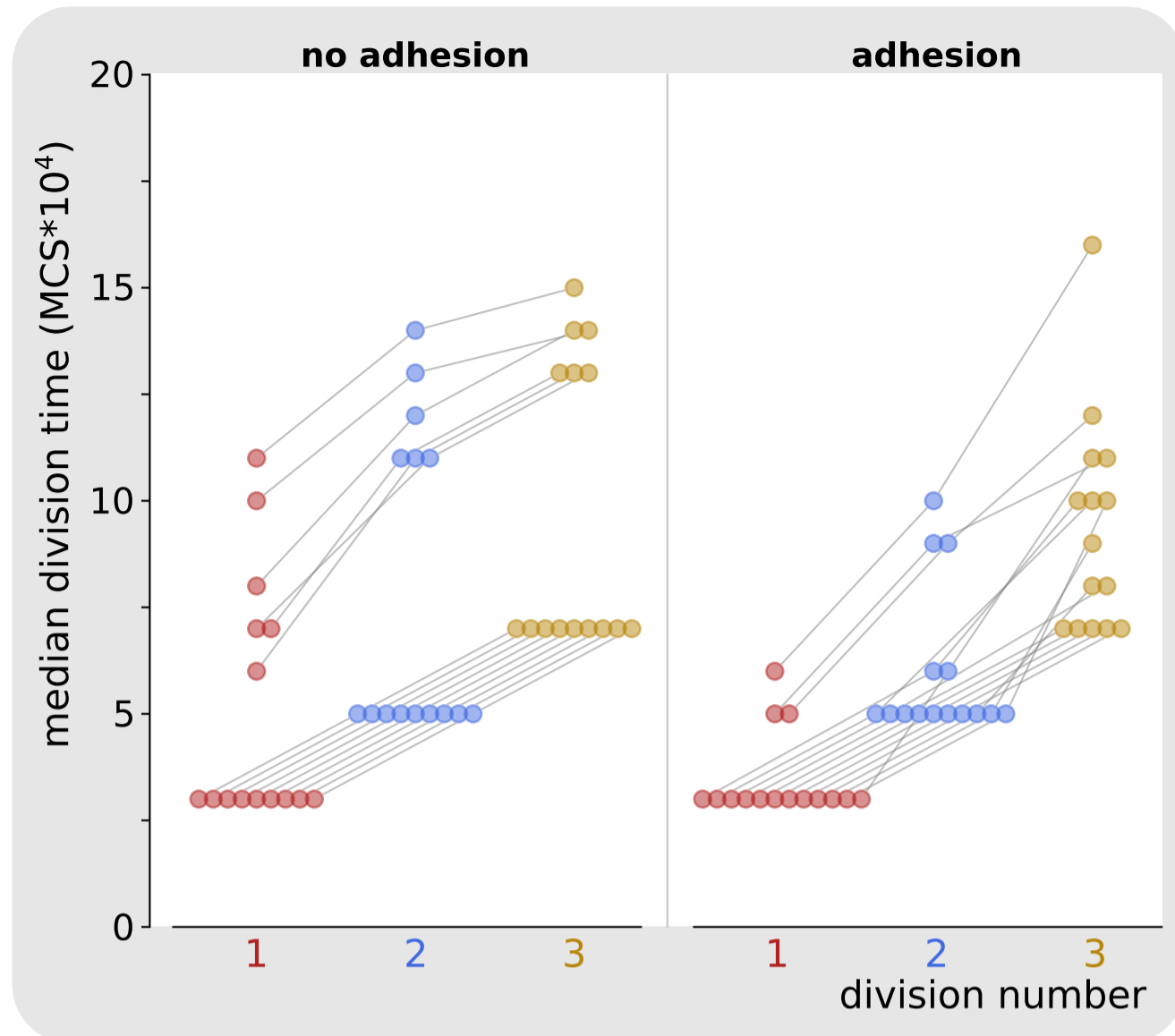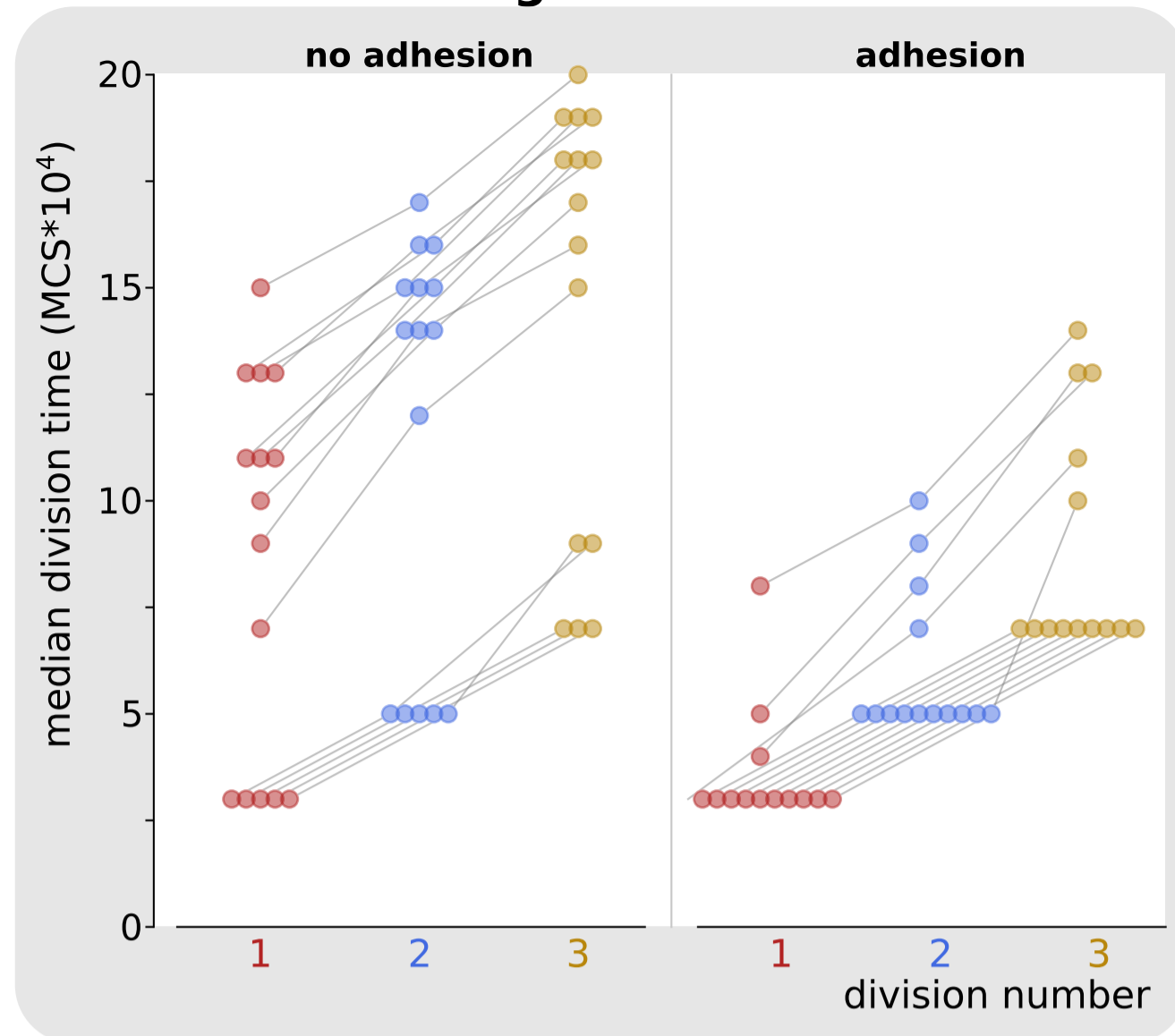

season=250000 MCS

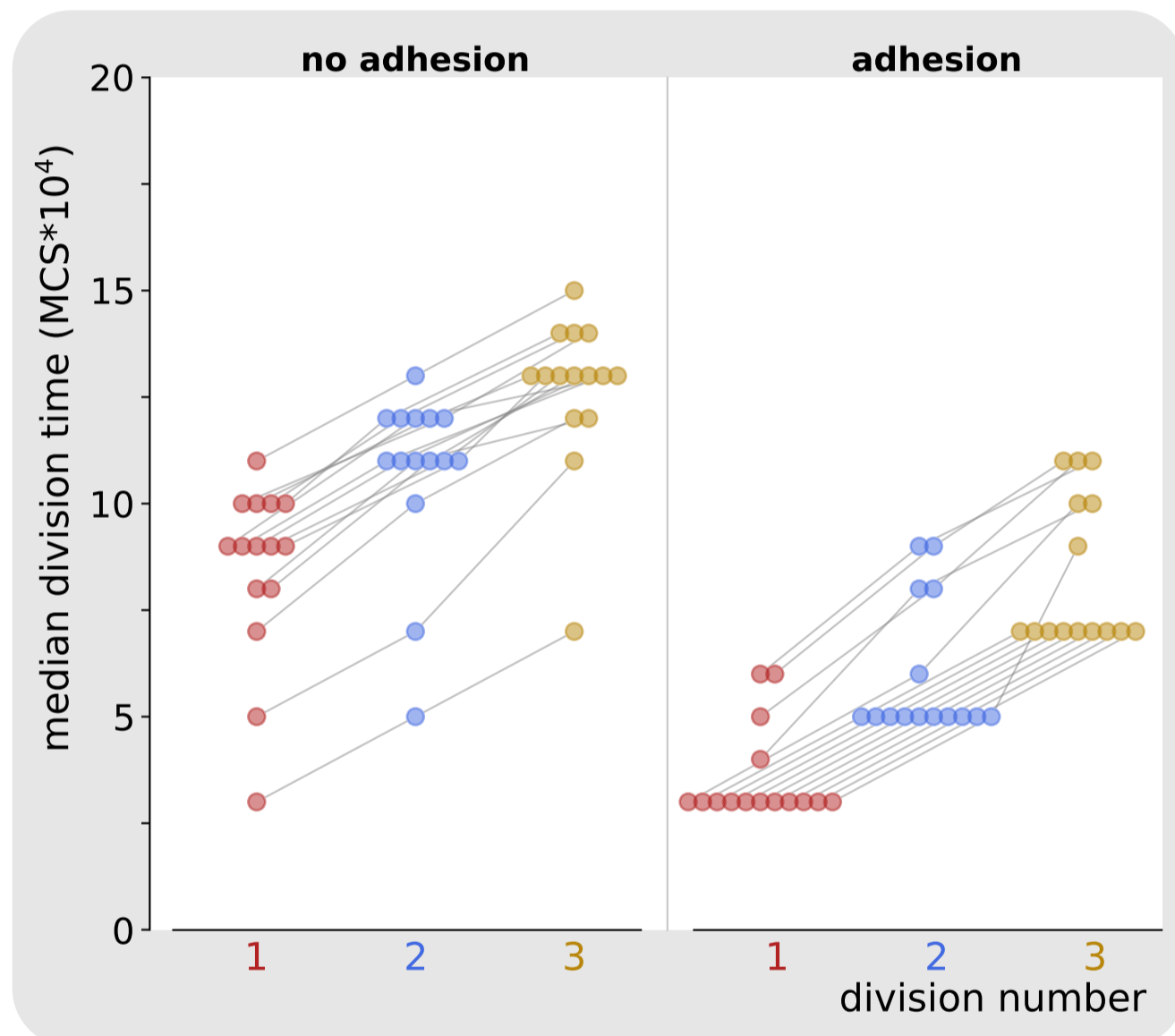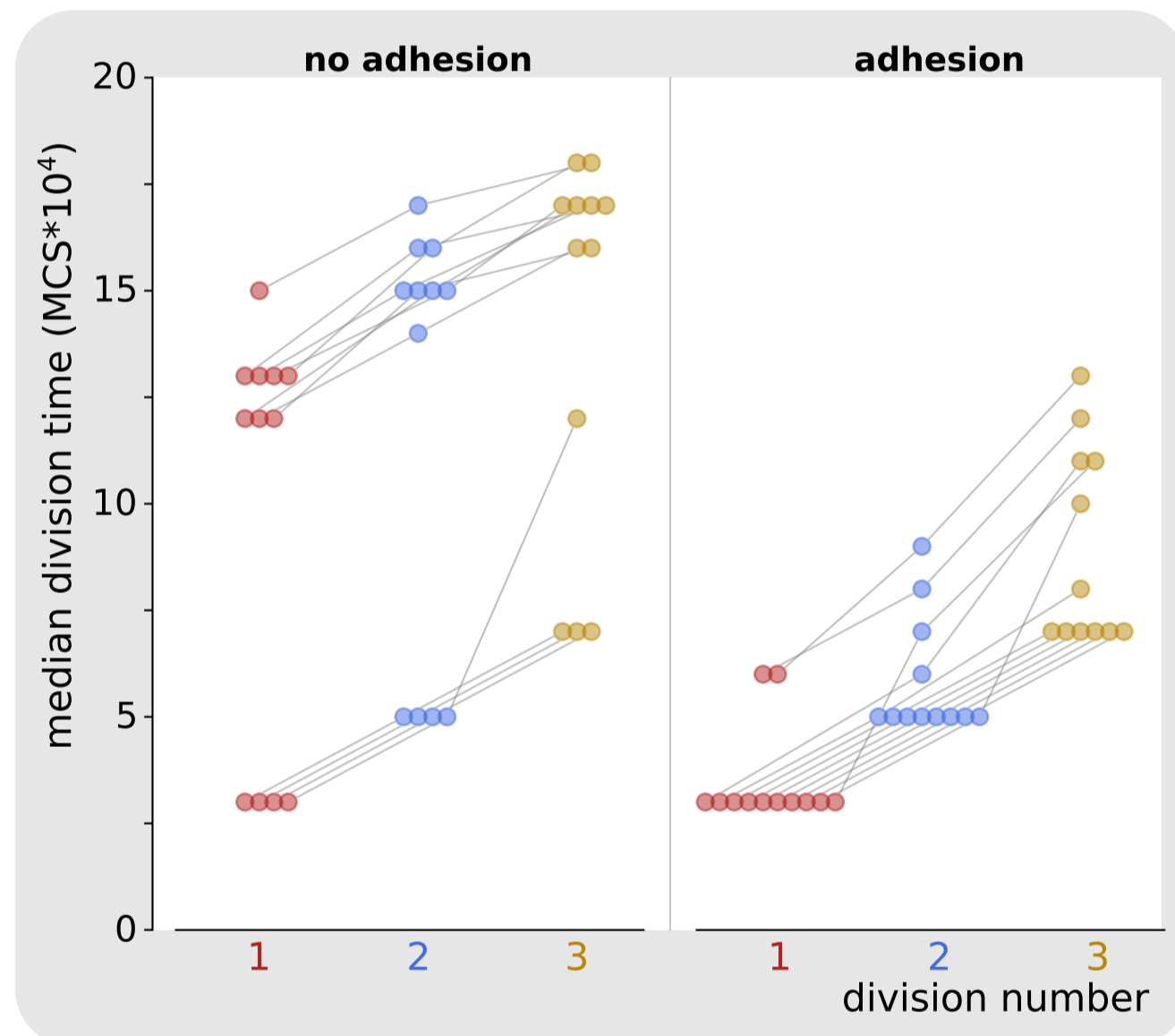

season=180000 MCS

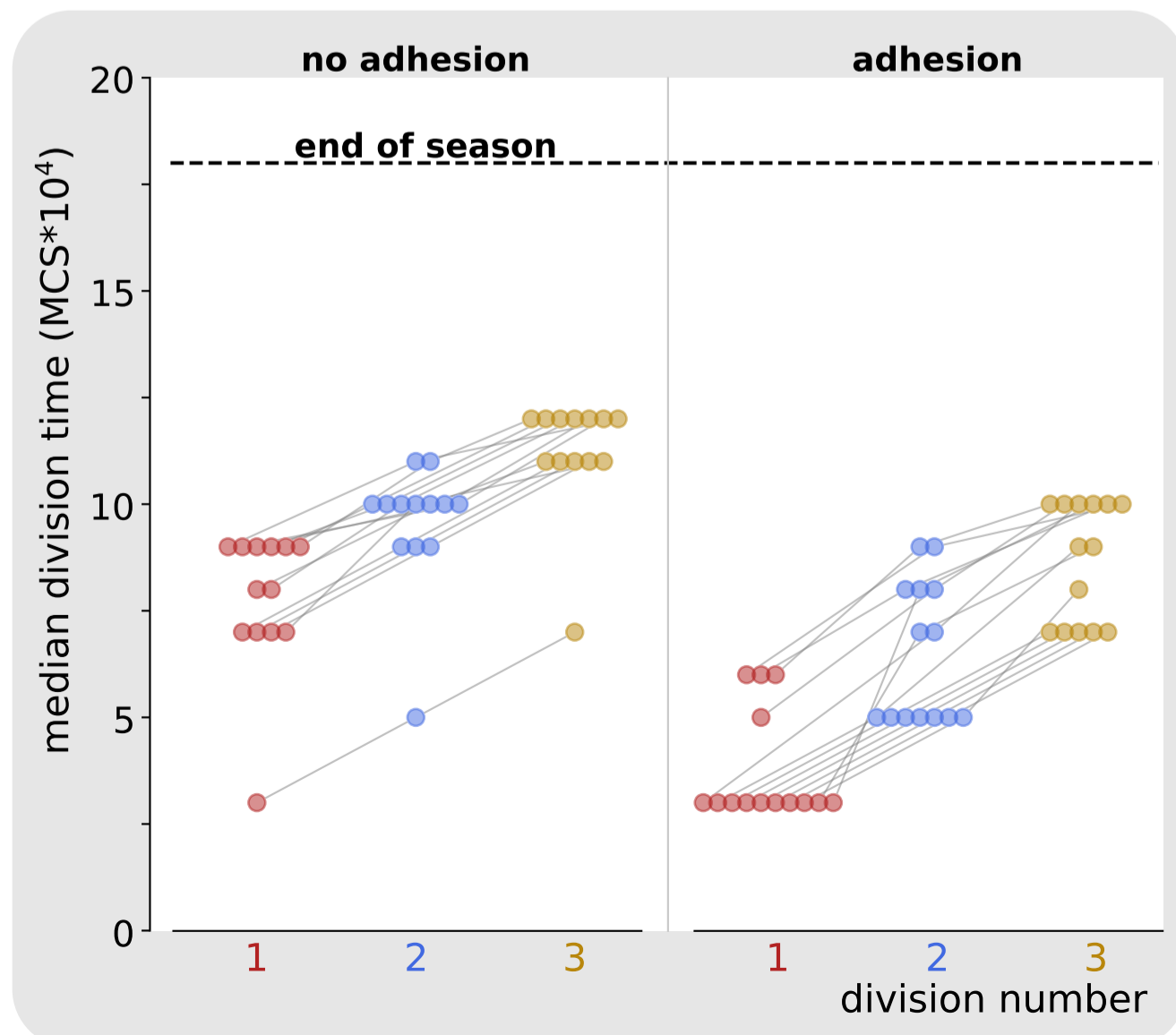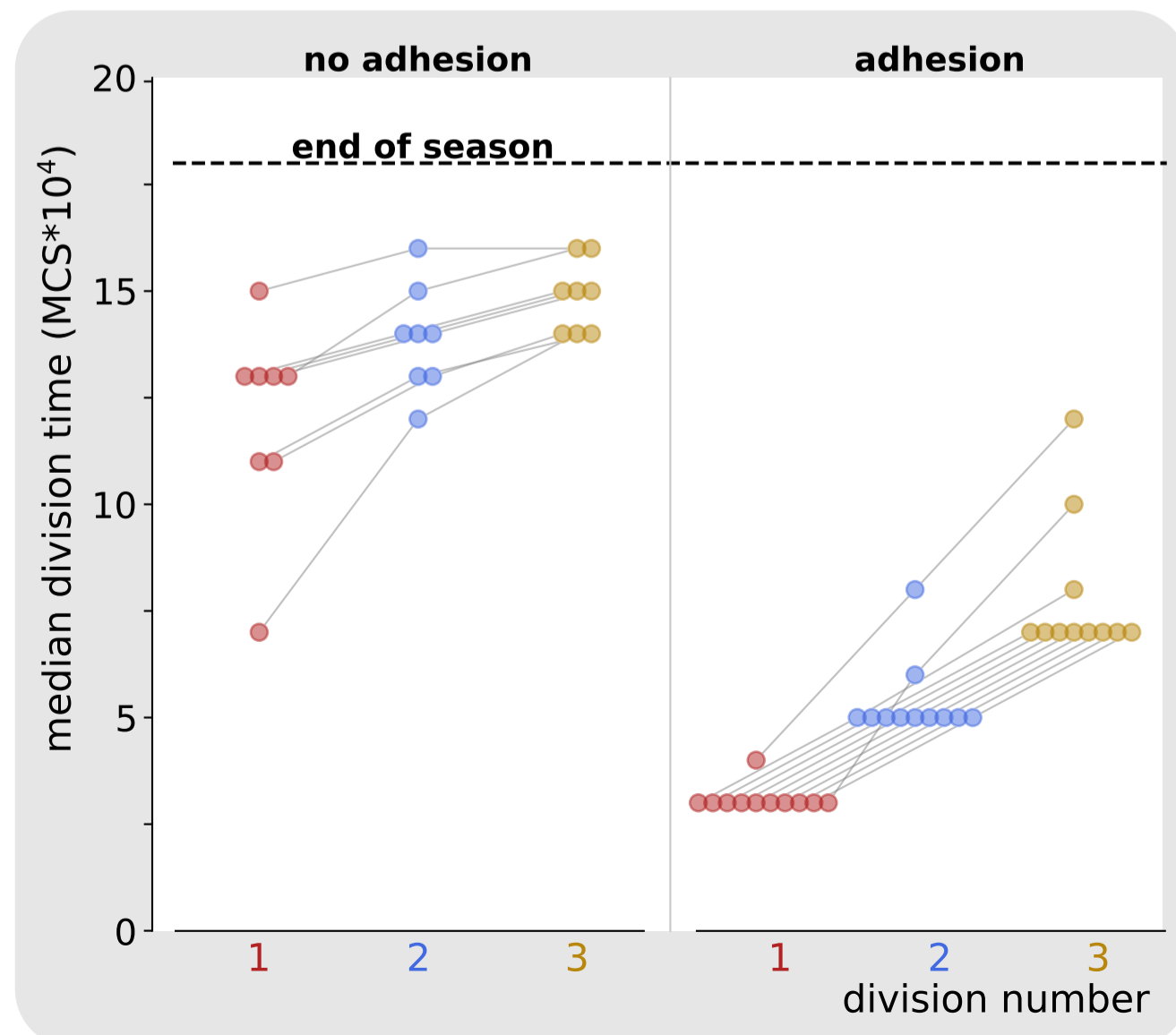

Supplement: Supplementary file 7 — Additional file 7. Evolved division timing strategies in all simulation sets. The median timing of cell divisions of the last 10 seasons in all sets of simulations, with varying season duration, death rates and with or without evolution of adhesion. Lines between dots connect values belonging to the same simulations. [file 12862_2023_2133_MOESM7_ESM.pdf]

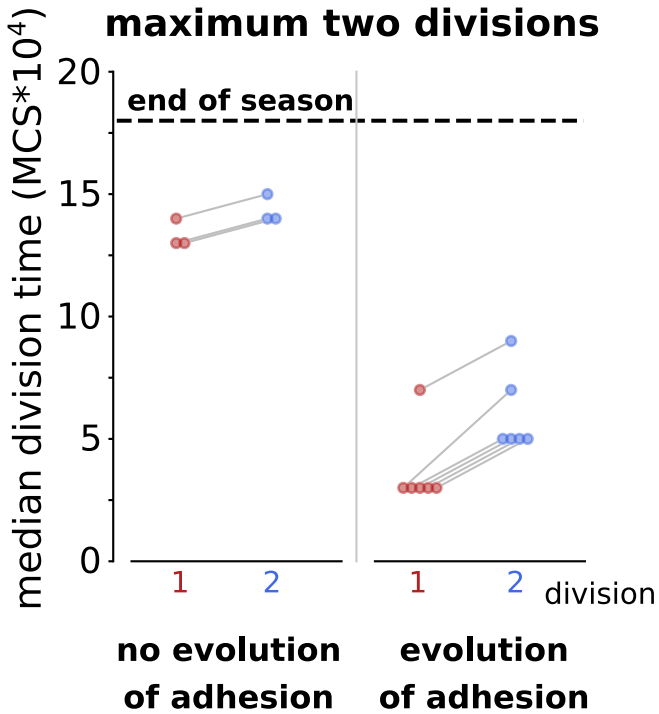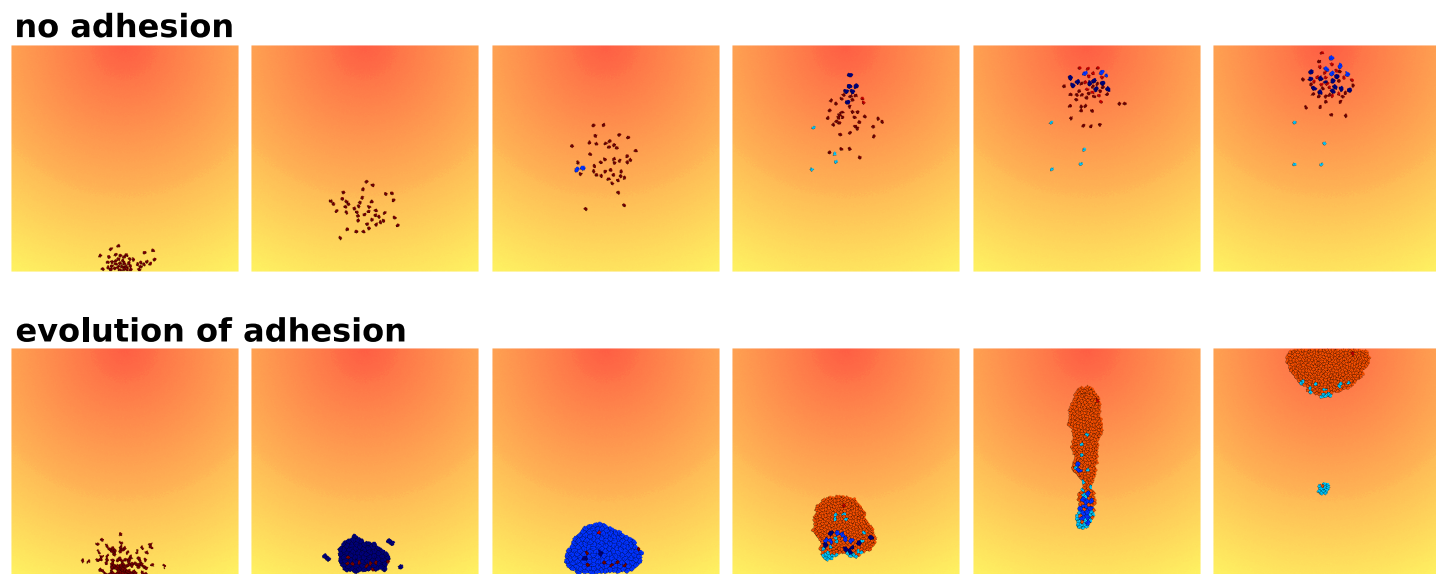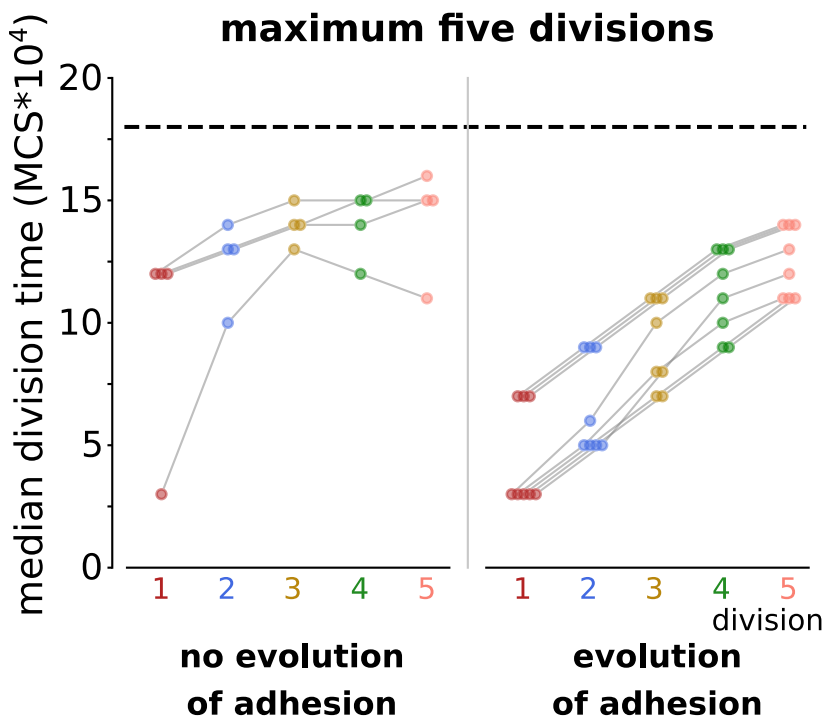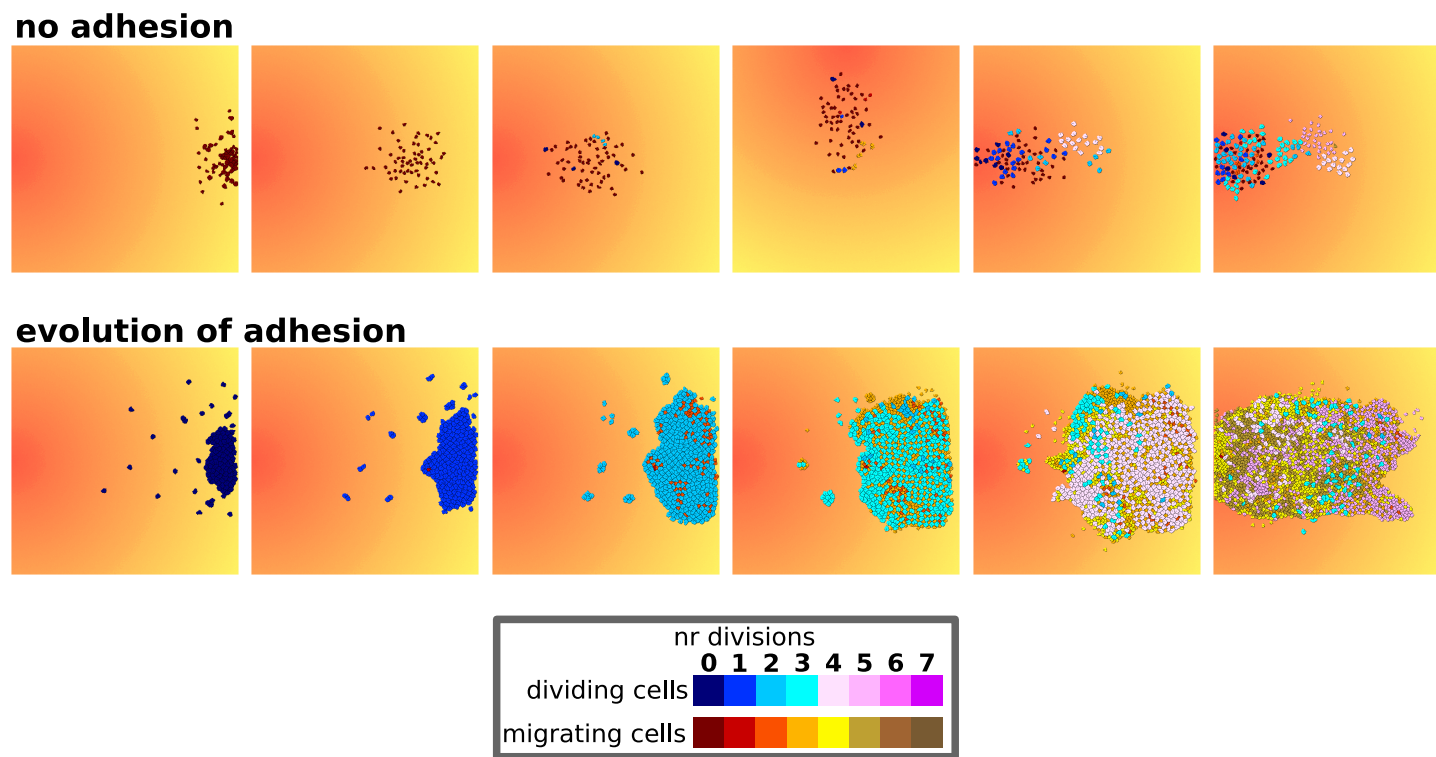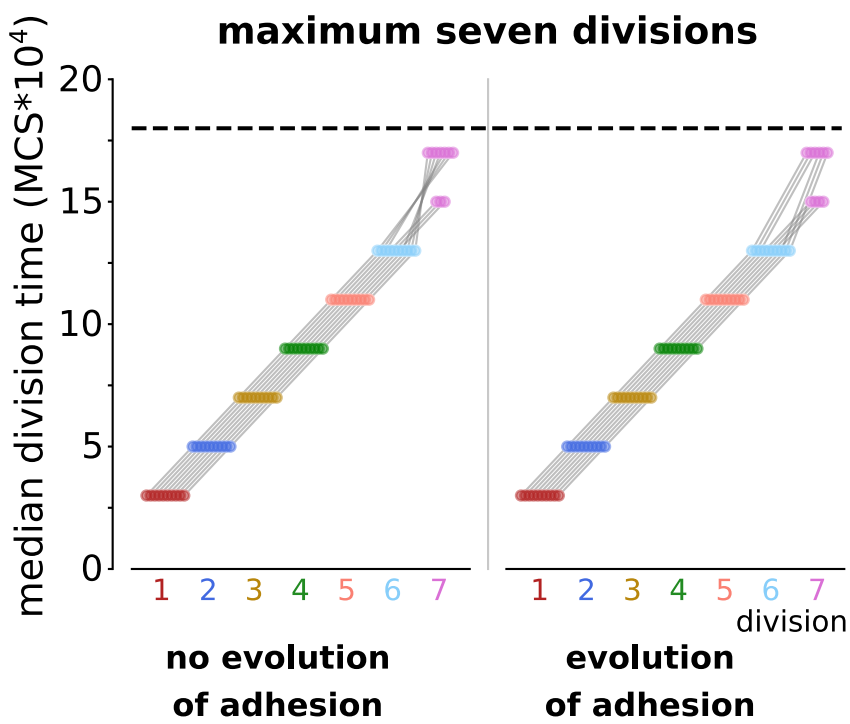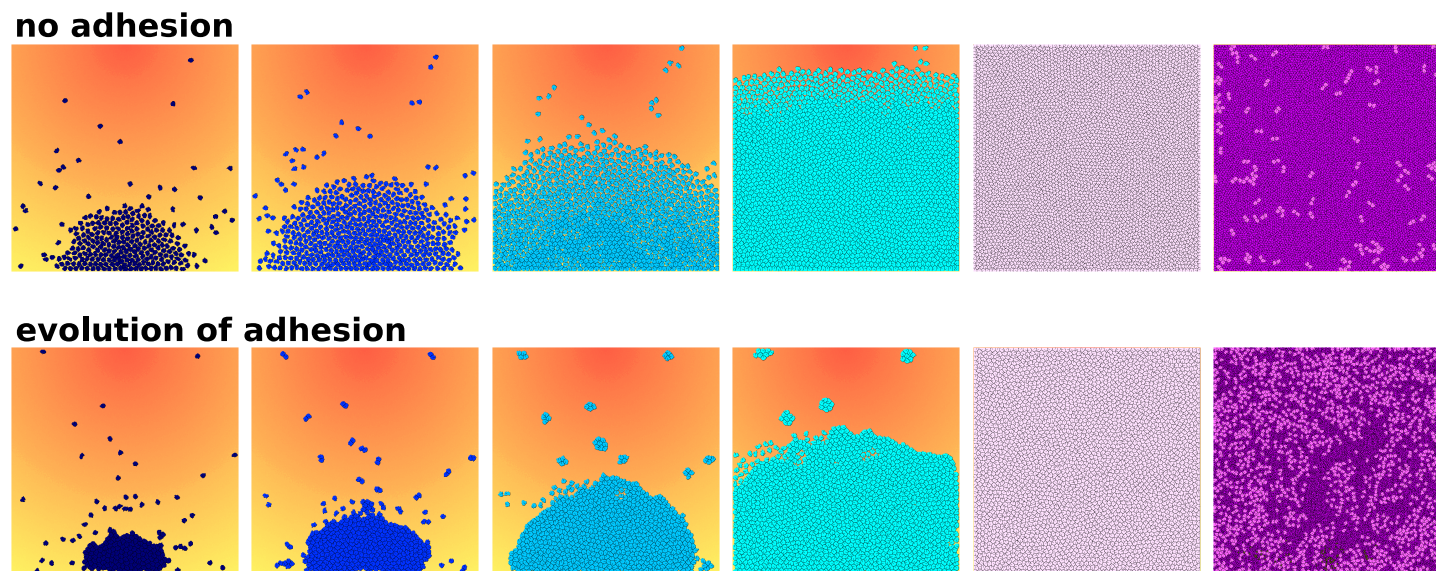

Supplement: Supplementary file 8 — Additional file 8. Evolved division timing strategies in simulations with a different maximum number of divisions. The median timing of cell divisions of the last 10 seasons in simulations with high selection pressure (short seasons, 180 000 MCS; and high death probability, $d_{scale} = 40$dscale=40). Lines between dots connect values belonging to the same simulations. On the right, snapshots of one season in representative simulations are displayed. [file 12862_2023_2133_MOESM8_ESM.pdf]

**A**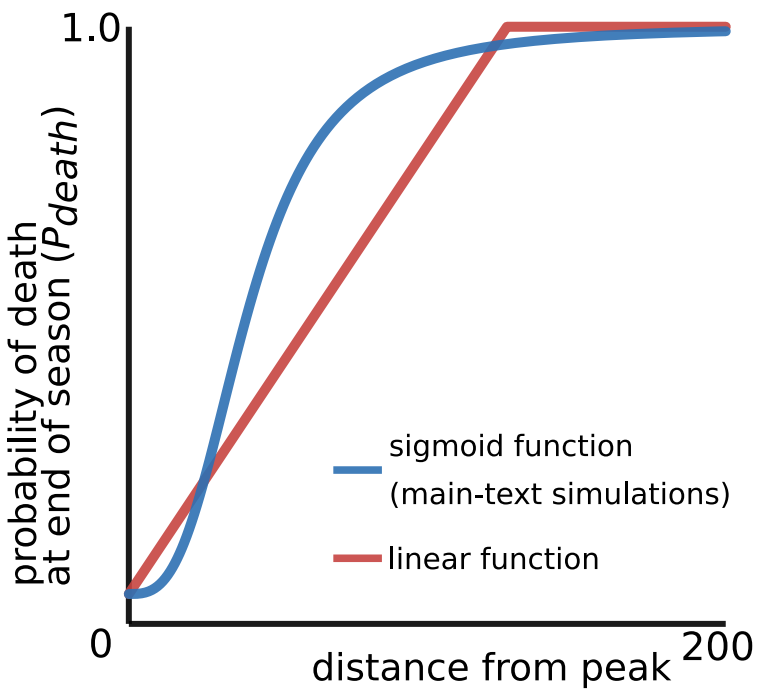**B**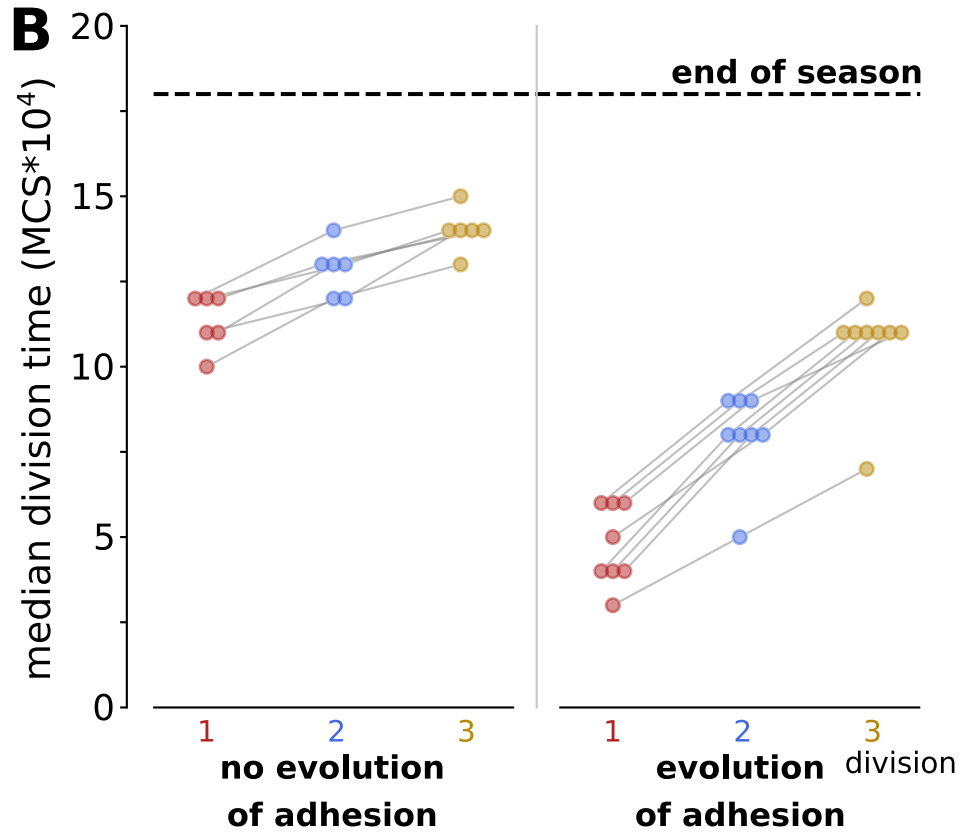**C****no adhesion**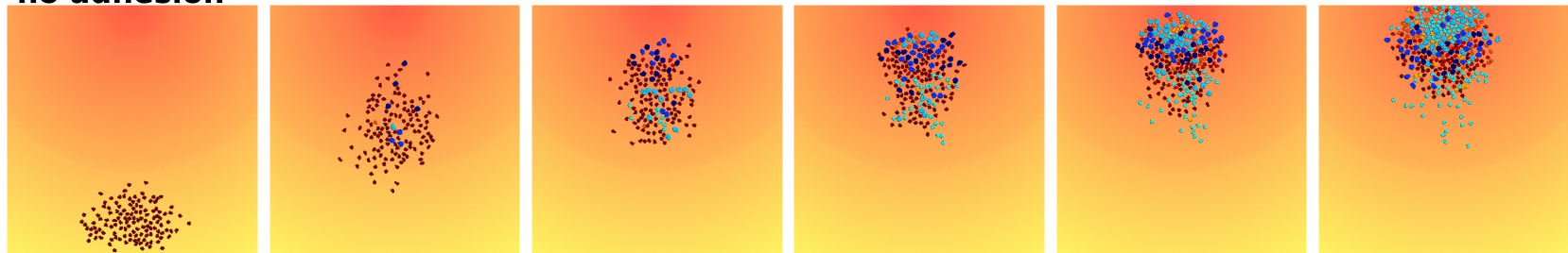**evolution of adhesion**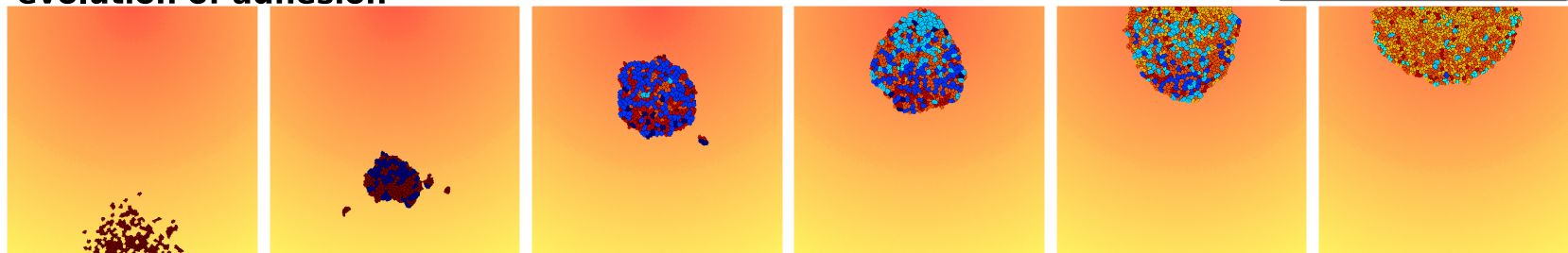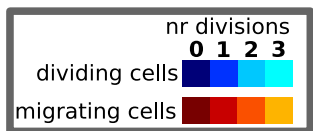

Supplement: Supplementary file 9 — Additional file 9. Evolved division timing strategies in simulations with different death function. A) The function that determines the probability of death at the end of the season based on the distance of a cell from the peak of the gradient. In blue the function used for the simulations in the main text; in red, a linear function that a linear function that increases until it reaches probability of death = 1, and remains constant for further distances. B) Simulations run with or without adhesion, using the linear death rate function. C) Snapshots from one season of representative simulations with a linear death rate. [file 12862_2023_2133_MOESM9_ESM.pdf]

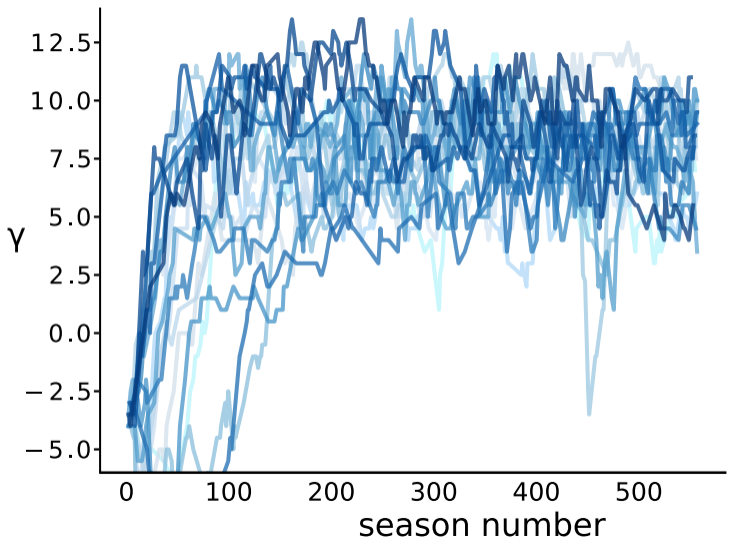

Supplement: Supplementary file 10 — Additional file 10. Evolution of adhesion in simulations starting from individual evolved without adhesion. Evolution of adhesion in 20 simulations, started with 4 individuals that had evolved without adhesion (5 independent simulations per individual). [file 12862_2023_2133_MOESM10_ESM.pdf]

**A**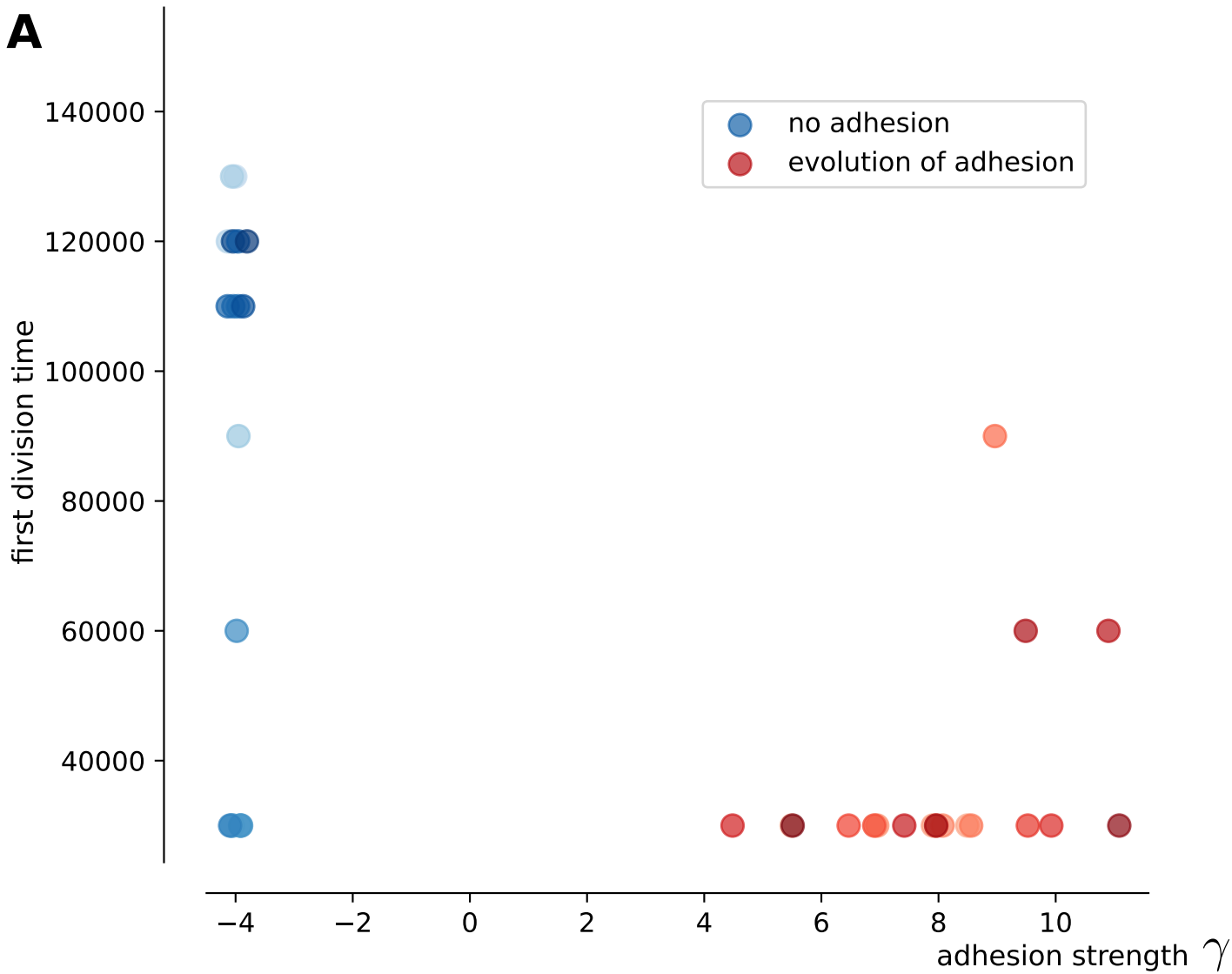**B**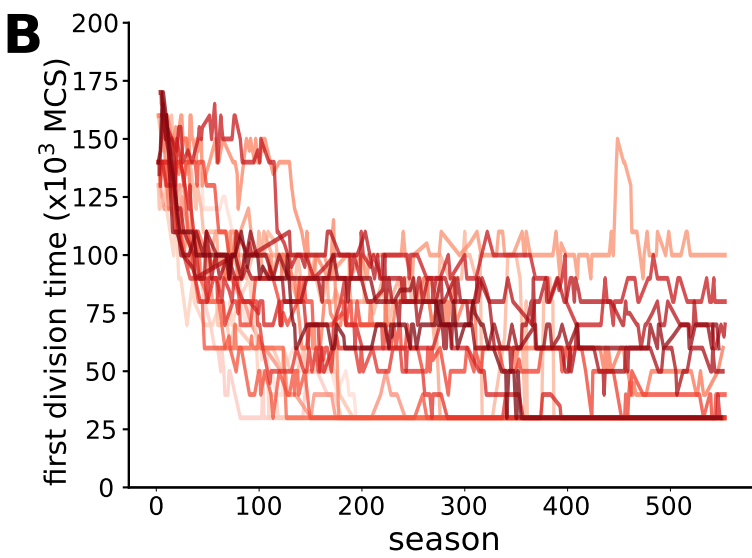**B'**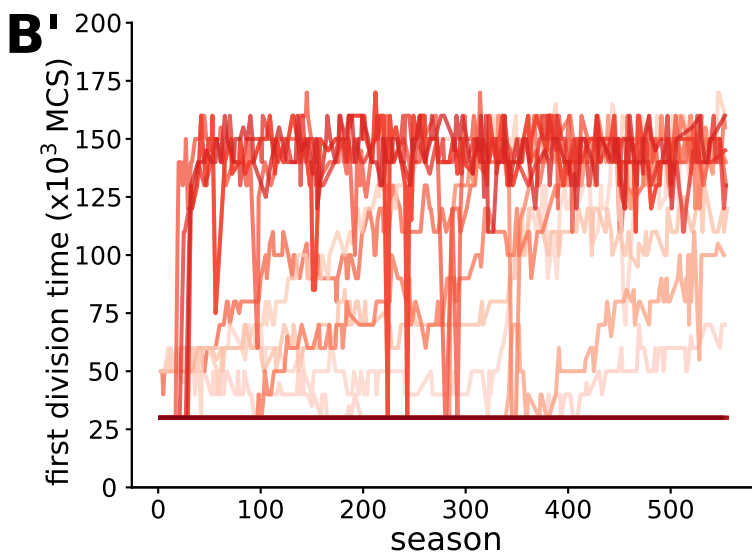

Supplement: Supplementary file 11 — Additional file 11. Evolution of the opposite cell regulation strategy when adhesion is switched. A) For all simulations, the evolved timing of the first division is plotted against the evolved adhesion strength. Blue: simulations that were started with an adhering ancestor, but continued evolution without adhesion ($\gamma$γ fixed to -4); red: simulations started with a non-adhering ancestor that were allowed to evolve adhesion. B) Evolution of division timing in simulations started with a non-adhering ancestor. B’) Evolution of division timing over multiple seasons in simulations started with an adhering ancestor that were continued without adhesion. [file 12862_2023_2133_MOESM11_ESM.pdf]

**division-late strategy**

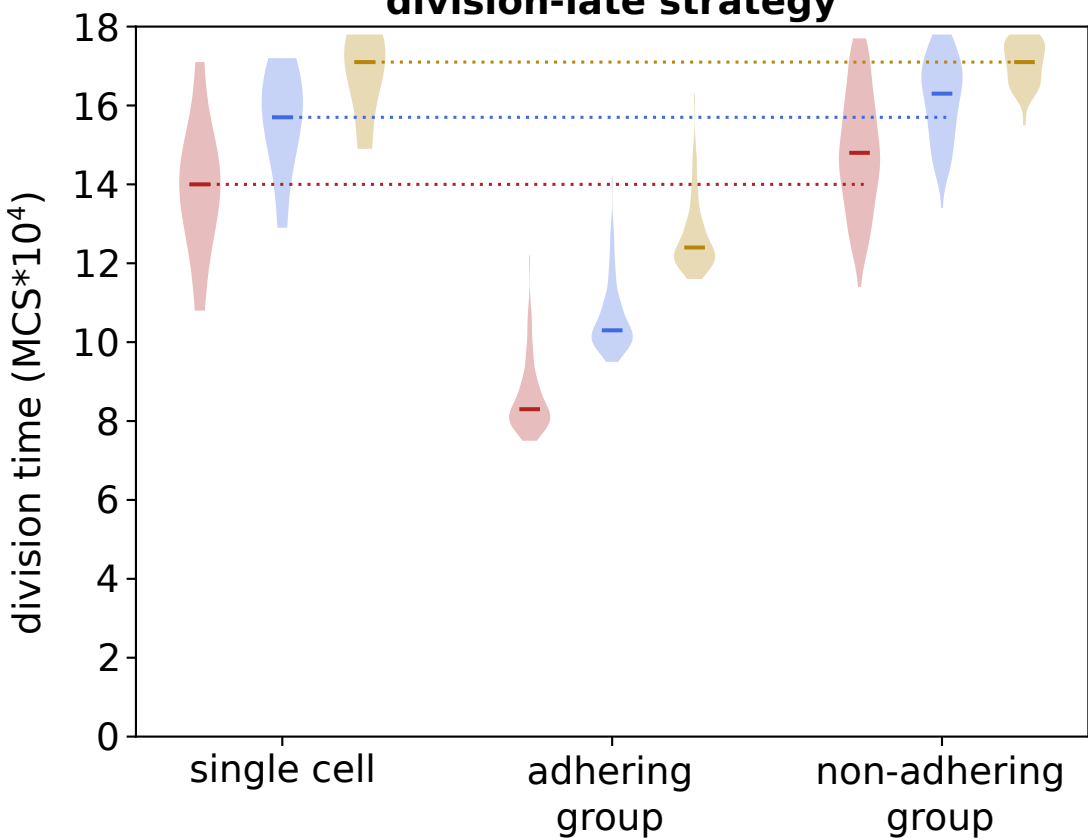

**division-early strategy**

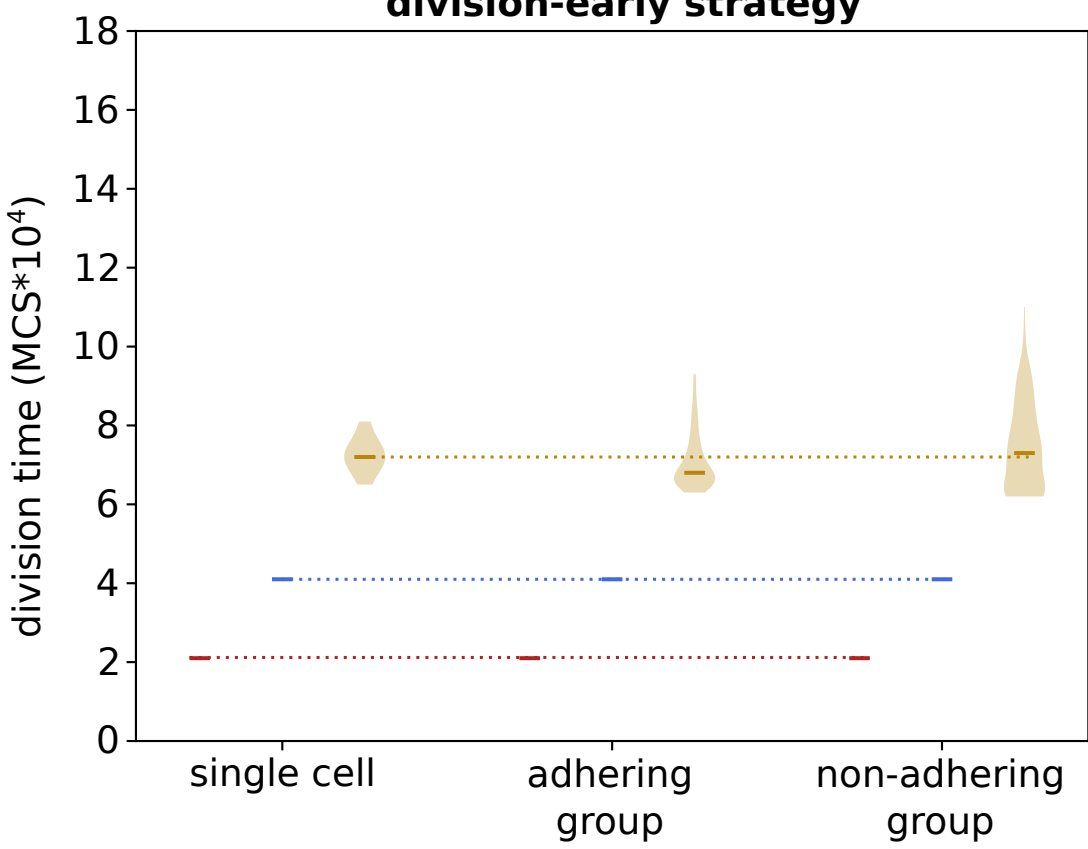

Supplement: Supplementary file 12 — Additional file 12. Effect of adhesion or non-adhesion on division timing. The distribution of division timings over one season for simulations with a single cell compared to simulations with 20 adhering or non-adhering cells with identical regulatory networks. For simplicity, divisions were simulated by having the cell cease migration or the same amount of time as an actual division would last, but without creating an additional daughter cell. This kept the group size the same throughout the run. There were also no mutations of adhesion or regulation. We show here two examples, one with cells evolved without adhesion, possessing a division-late strategy; and one with cells evolved with adhesion, possessing a division-early strategy. Season duration=180000 MCS. [file 12862_2023_2133_MOESM12_ESM.pdf]

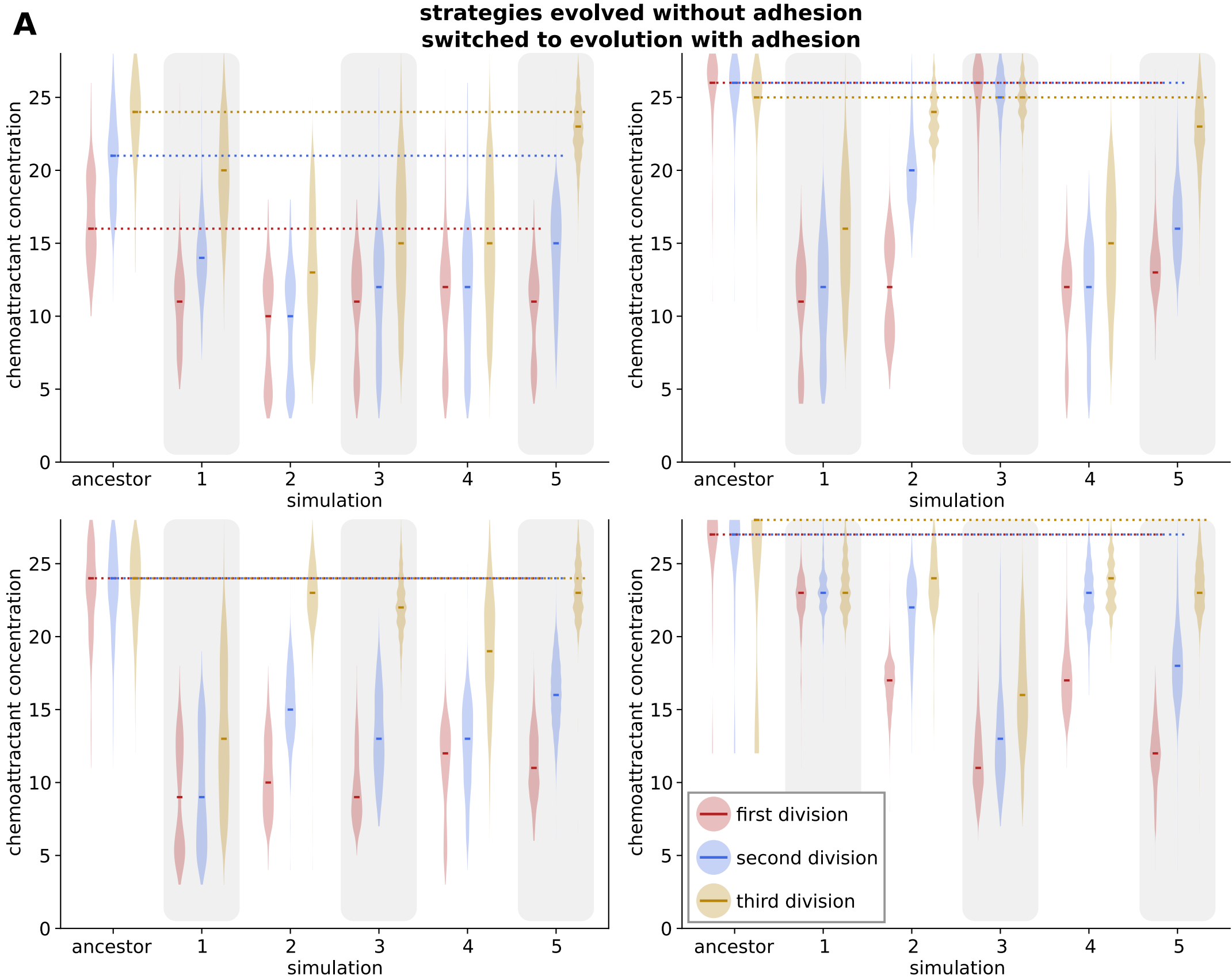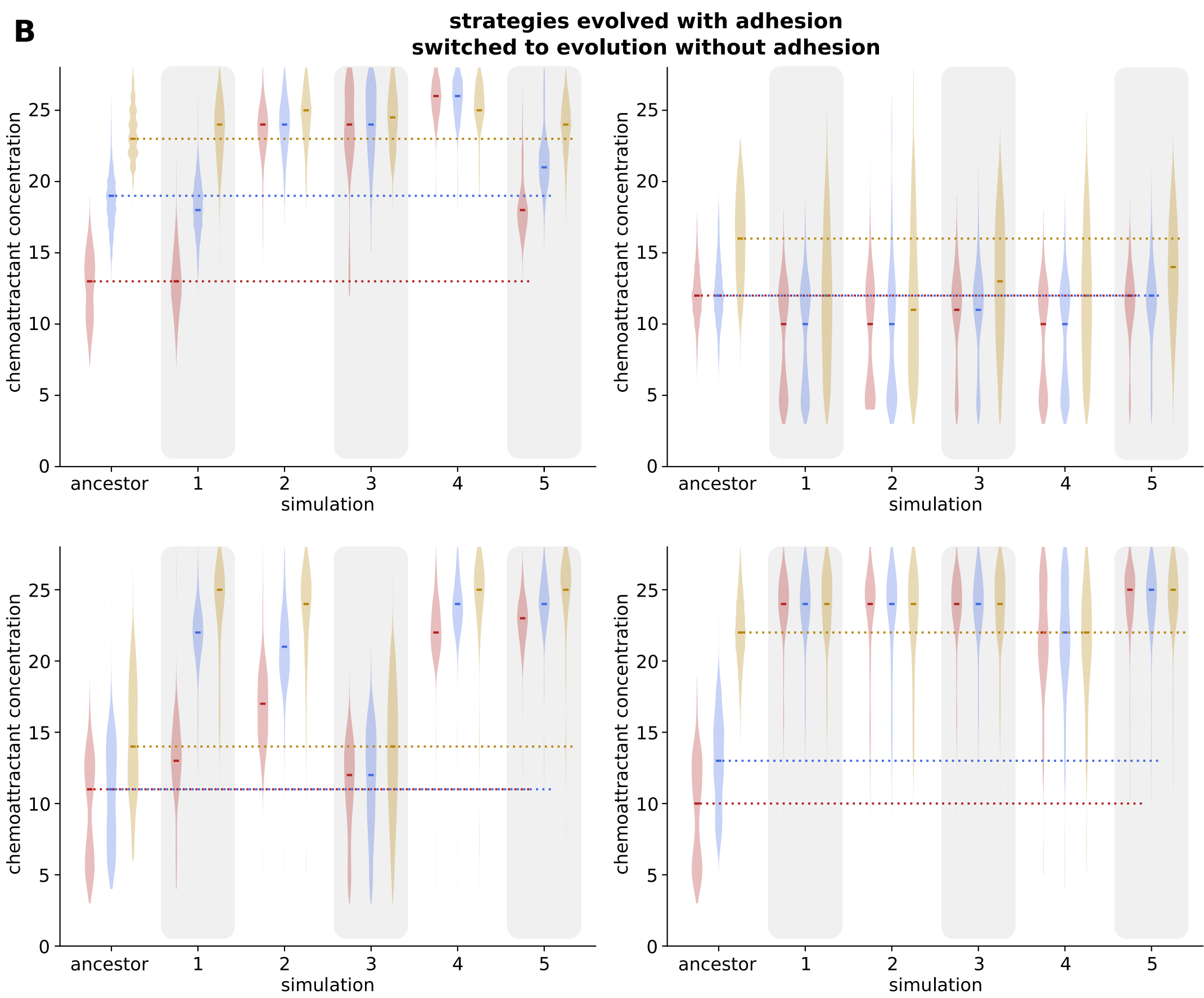

Supplement: Supplementary file 13 — Additional file 13. Evolution of gradient sensing in populations switched to opposite adhesion regime. Distributions of the chemoattractant concentration at which divisions happen, in the ancestor and the five replicate simulations evolved from that ancestor. A) Simulations seeded with individuals evolved without adhesion, continued with evolution of adhesion. B) Simulations seeded with individuals evolved with adhesion, continued without adhesion. [file 12862_2023_2133_MOESM13_ESM.pdf]

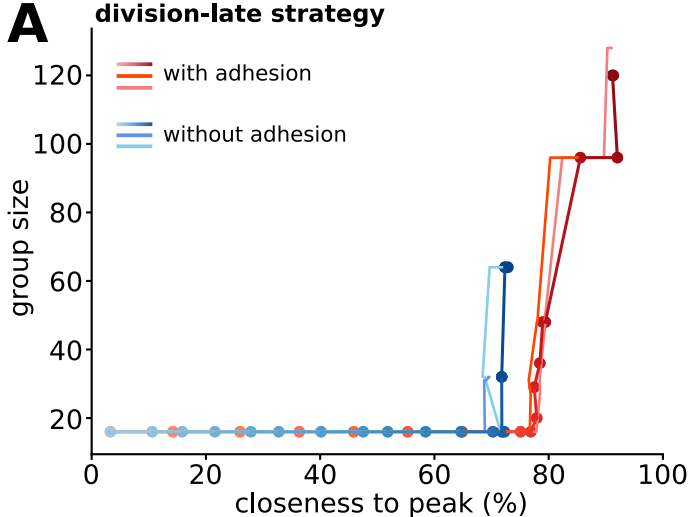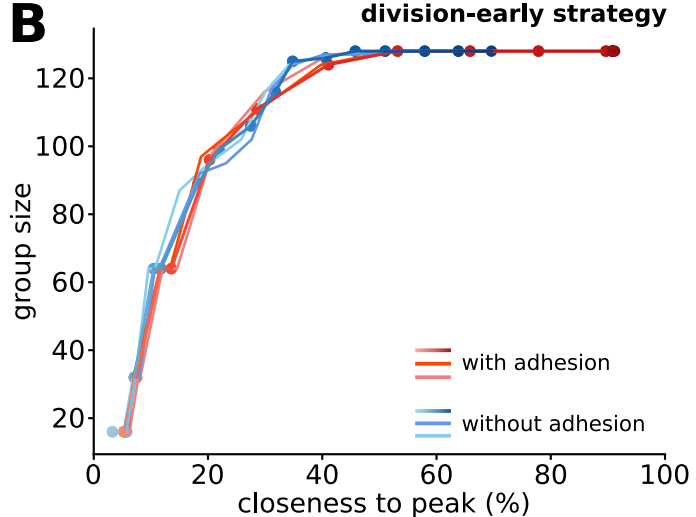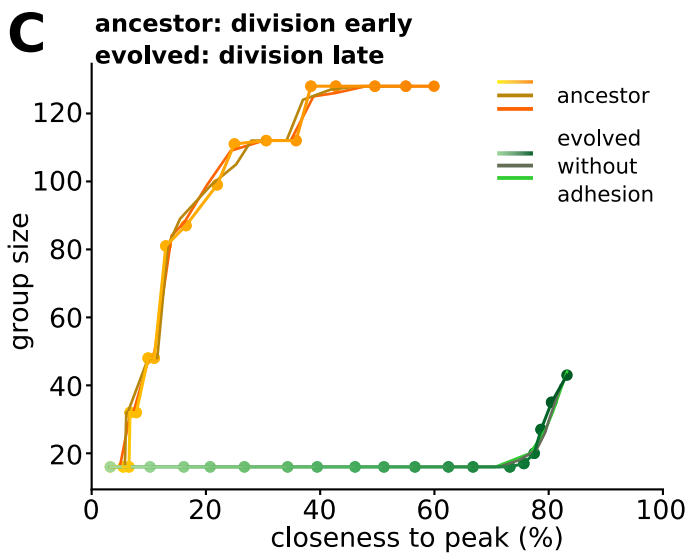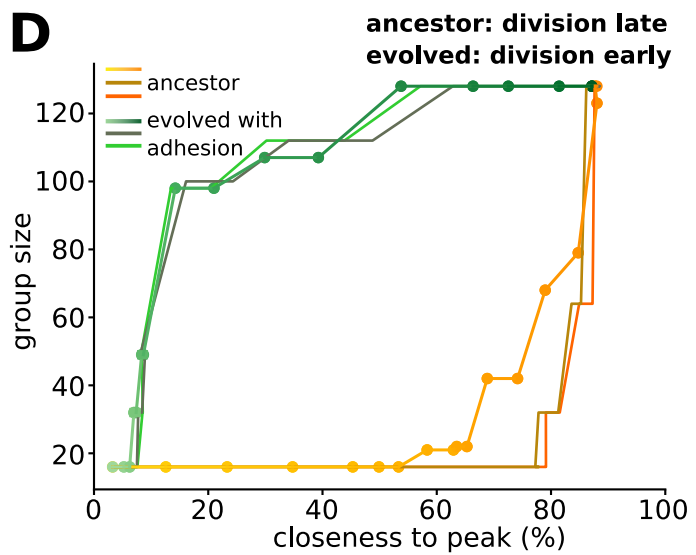

Supplement: Supplementary file 17 — Additional file 17. Replicates of competition experiment. Group size plotted against the median distance of cells to the peak of gradient, shown for the first season of the competition experiment (0%=maximum distance, 100%=at the peak). The graded lines with dots are the same as in Fig. 4. a-b) Competition between two groups with the same regulatory strategy, one adhering ($\gamma = 6$γ=6; a), the other non-adhering ($\gamma = -4$γ=-4; b). c) Competition between ancestral, division-early strategy and evolved, division-late strategy; both non-adhering. d) Competition between two adhering groups, one with an ancestral, division-late strategy and one with a strategy evolved with adhesion (having become more division-early). [file 12862_2023_2133_MOESM17_ESM.pdf]
